# Targeting Patients’ Cognitive Load for Telehealth Video Visits Through Student-Delivered Helping Sessions at a United States Federally Qualified Health Center: Equity-Focused, Mixed Methods Pilot Intervention Study

**DOI:** 10.2196/42586

**Published:** 2023-02-01

**Authors:** Marcy G Antonio, Alicia Williamson, Vaishnav Kameswaran, Ashley Beals, Elizabeth Ankrah, Shannon Goulet, Yucen Wang, Grecia Macias, Jade James-Gist, Lindsay K Brown, Sage Davis, Srijanani Pillai, Lorraine Buis, Tawanna Dillahunt, Tiffany C Veinot

**Affiliations:** 1 School of Information University of Michigan Ann Arbor, MI United States; 2 Department of Informatics University of California Irvine Irvine, CA United States; 3 School of Public Health University of Michigan Ann Arbor, MI United States; 4 Covenant Community Care Detroit, MI United States; 5 Department of Family Medicine University of Michigan Ann Arbor, MI United States; 6 School of Information and College of Engineering University of Michigan Ann Arbor, MI United States; 7 Department of Health Behavior and Health Education School of Information and School of Public Health University of Michigan Ann Arbor, MI United States

**Keywords:** COVID-19, cognitive load, health services accessibility, health equity, human-computer interaction, pilot projects, learning, telemedicine and telehealth, recruit methods, digital health intervention, patient portal, video consultation, mobile phone

## Abstract

**Background:**

The task complexity involved in connecting to telehealth video visits may disproportionately impact health care access in populations already experiencing inequities. Human intermediaries can be a strategy for addressing health care access disparities by acting as *technology helpers* to reduce the cognitive load demands required to learn and use patient-facing telehealth technologies.

**Objective:**

We conducted a cognitive load theory–informed pilot intervention involving *warm accompaniment* telehealth helping sessions with patients at a Federally Qualified Health Center (FQHC). We demonstrate how to design and report recruitment methods, reach, delivery process, and the preliminary impact of a novel equity-focused intervention.

**Methods:**

Early into the COVID-19 pandemic a telehealth helping session was offered to patients at FQHC via phone. Graduate students led the sessions on conducting a telehealth video test run or helping with patient portal log-in. They systematically recorded their recruitment efforts, intervention observations, and daily reflection notes. Following the intervention, we asked the intervention participants to participate in an interview and all patients who had telehealth visits during and 4 weeks before and after the intervention period to complete a survey. Electronic health records were reviewed to assess telehealth visit format changes. Descriptive and inferential statistical analyses of the recruitment records, electronic health record data, and surveys were performed. Through integrative analysis, we developed process-related themes and recommendations for future equity-focused telehealth interventions.

**Results:**

Of the 239 eligible patients, 34 (14.2%) completed the intervention and 3 (1.2%) completed subsequent interviews. The intervention participants who completed the survey (n=15) had lower education and less technological experience than the nonintervention survey participants (n=113). We identified 3 helping strategies for cognitive load reduction: *providing step-by-step guidance for configuring and learning*, *building rapport to create confidence while problem-solving*, and *being on the same page to counter informational distractions*. Intervention participants reported increased understanding but found that learning the video visit software was more difficult than nonintervention participants. A comparison of visit experiences did not find differences in difficulty (cognitive load measure) using telehealth-related technologies, changes to visit modality, or reported technical problems during the visit. However, the intervention participants were significantly less satisfied with the video visits.

**Conclusions:**

Although a limited number of people participated in the intervention, it may have reached individuals more likely to need technology assistance. We postulate that significant differences between intervention and nonintervention participants were rooted in baseline differences between the groups’ education level, technology experience, and technology use frequency; however, small sample sizes limit conclusions. The barriers encountered during the intervention suggest that patients at FQHC may require both improved access to web-based technologies and human intermediary support to make telehealth video visits feasible. Future large, randomized, equity-focused studies should investigate blended strategies to facilitate video visit access.

## Introduction

### Background

The unprecedented uptake of telehealth services during the COVID-19 pandemic [1] may have amplified disparities in health care access [2,3], as demonstrated by the different adoption rates for the modality of telehealth visits. People with limited English proficiency [3-5] or lower socioeconomic status [2,4,6,7], older adults [5-9], and Black and Hispanic individuals [5-10] are more likely to have telehealth visits conducted over the phone (vs video). As such, these populations may be denied opportunities to share and access beneficial visual information communicated through video visits. Few studies have focused on methods to increase telehealth video visit uptake in low-resourced contexts, such as Federally Qualified Health Center (FQHC) and primarily their patients with low income. Previous strategies for improving the uptake of patient-facing technologies (eg, patient portals) for underserved populations often involved in-person professional assistance [11]—something that was not possible during the early stages of the COVID-19 pandemic and may not be feasible again in the face of future public health crises.

For patients to successfully conduct a telehealth video visit, they must have access to up-to-date devices and broadband internet [4,8,10,12-15], be able to afford plans with sufficient data [14], and have opportunities to develop necessary digital skills [16,17]. Widening technology and health disparities are often attributed to these access issues. However, less attention has been given to the relationship between task complexity and health disparities. Specifically, technologies that add to the complexity of tasks and processes may widen disparities [18], whereas technologies that simplify processes may help narrow the disparity gap [18,19]. Considerations for complexity and equity have become even more critical over the past 3 years, as the tasks and processes introduced with the rapid implementation of telehealth may have added to the cognitive load required when interacting with patient-facing technologies and completing patient health-related activities [20-22].

### Prior Work

#### Cognitive Load Reduction

Equitable telehealth access requires reducing the cognitive load introduced by the complex tasks involved in conducting telehealth video visits. Cognitive load is the effort and mental resources required to complete a task [22]. Task-related factors that introduce complexity and impact cognitive load include (1) the novelty and structure of the task, (2) the number of items that need to be learned and processed (ie, “elements”), (3) interactions between these elements, (4) switches between different platforms or screens (ie, “context-switching”), (5) extraneous information introduced through the environment, and (6) a person’s cognitive capacity and prior skills [22-26].

Previous research has demonstrated that psychological stress can tax one’s cognitive load and emotional resources [27-29]. In addition, stressors experienced in the face of illness and poverty may negatively impact engagement with complex tasks and learning processes for using technologies. The COVID-19 pandemic may have exasperated the baseline impact on cognitive load because of the amplified stressors that patients at FQHC experience through increased housing, job, and food security challenges [30-32]. Therefore, we posited that equity-focused interventions involving human intermediaries might help reduce the cognitive load of telehealth video visits.

#### Human Intermediaries as Technology Helpers

*Warm accompaniment* human intermediaries are one potential equity-focused strategy to reduce cognitive load. Human intermediaries act in a “middle space” between people and technologies, enabling novice users to “locate, retrieve, understand, cope with, and use” patient-facing health information technologies [33]. *Warm experts* [34] are a type of intermediary that focuses on guiding through complex digital ecosystems while also supporting the emotional comfort of novice technology users [35,36].

The COVID-19 pandemic has spurred an increased uptake of intermediaries who serve as “digital navigators” for telehealth technologies [37-39]. Notably, models for intermediary services differ in terms of (1) *who provides* support (eg, staff, trained volunteers, peers, family, or friends), (2) *who receives* the support (eg, older adults or people with limited economic resources), and (3) *what types* of support are provided (eg, navigation, troubleshooting, or in-depth training) [14,37-39]. From an FQHC context, it may be financially infeasible to have staff or community health workers provide technology intermediation. Accordingly, we investigated a model in which trained graduate students, onboarded as FQHC volunteers, could provide one-time *helping sessions* to support patients in preparing for upcoming telehealth video visits. In line with other recent work [39], we posited that having community engagement projects in which students conduct helping sessions as part of their training could establish a sustainable intermediary model after the pilot period.

We extend prior telehealth and intermediary work [14,37-39] by piloting an intervention in which *warm accompaniment* facilitated learning, helped reduce patients’ cognitive load, and minimized barriers to telehealth video visits. Furthermore, in contrast to prior work [39], our equity-focused intervention deliberately targets *all* patients at an FQHC to assess recruitment strategies and reach underrepresented populations.

### Objectives

We conducted a cognitive load theory–informed pilot intervention involving *warm accompaniment* telehealth helping sessions with patients at an FQHC. We demonstrate how to design, evaluate, and report on the recruitment methods, reach, delivery process, and preliminary impact [40,41] of a novel equity-focused intervention that facilitates access to patient portals and telehealth video visits for patients at FQHC. We investigated student-led intermediation strategies that could reduce the cognitive load of three tasks: (1) logging into the patient portal, (2) signing telehealth consent on the patient portal, and (3) conducting a telehealth video test run.

## Methods

### The Sociotechnical Context

The study’s participating FQHC offered telephone and video telehealth visits. However, the FQHC’s recent addition of multiple technologies introduced various novel, challenging tasks and processes for phone and video telehealth sessions. For instance, the FQHC’s new electronic health records (EHRs) vendors did not offer video visit capabilities at the beginning of the pandemic. To address this limitation, the FQHC adopted 2 telehealth platforms that supported video visits but lacked EHR integration. Notably, one vendor continues to only offer full video visit capabilities on iPhone Operating System devices, which tend to be adopted by higher-income populations [42,43].

In addition, the patients were asked to complete the telehealth consent form through their patient portal before their first telehealth visit. However, the FQHC had limited patient portal uptake owing to the transition to a new EHR system just 5 months before the COVID-19 pandemic: of the 9333 patients who had an appointment from October 2019 to March 13, 2020, 2311 (24.77%) had enrolled, 1134 (12.15%) had declined, and 418 (4.48%) had used the patient portal. Therefore, the FQHC recommended that in addition to the video test run, portal setup and telehealth consent completion be included in the intervention design, as they were both important tasks for telehealth video visits and novel to most patients at the FQHC.

Given the multiple patient-facing technologies involved in a telehealth video visit, the intermediary intervention focused on assisting patients with three tasks: (1) logging into the patient portal, (2) signing a telehealth consent form in the patient portal, and (3) testing telehealth video visits using a platform that was not integrated into the patient portal of FQHC.

### Methodological Foundations

We conducted a pilot study to develop and test the feasibility of an equity-focused telehealth intermediary intervention. Pilot studies are exploratory to inform the development of larger-scale randomized studies and do not strive for a large sample size that can be used for inferential statistics [41]. Recommendations for designing a pilot study include being theoretically informed, using diverse sampling across different qualitative methods, and having a comparator group to evaluate reach, recruitment methods, and preliminary impact [40,41,44].

Notably, our equity-focus design adds to the current intervention study approaches. Intervention designs often perpetuate the unjust allocation of resources and widen health disparities because of selection bias that favors the dominant population [45,46]. Toscos et al [47] emphasize the need to re-examine current approaches to technological intervention studies that lack the representation of people with limited technology backgrounds. Our study aimed to address this call through an equity-focus intervention, which we define as an approach that embraces generalizability toward underrepresented populations [47]. To illustrate the methodological approach for an equity-focused intervention, we detail process delivery strategies and recruitment methods for hard-to-reach populations, and “best-practice” for reporting results on health equity initiatives.

### Theoretical Foundations of the Intervention

**Figure 1:**
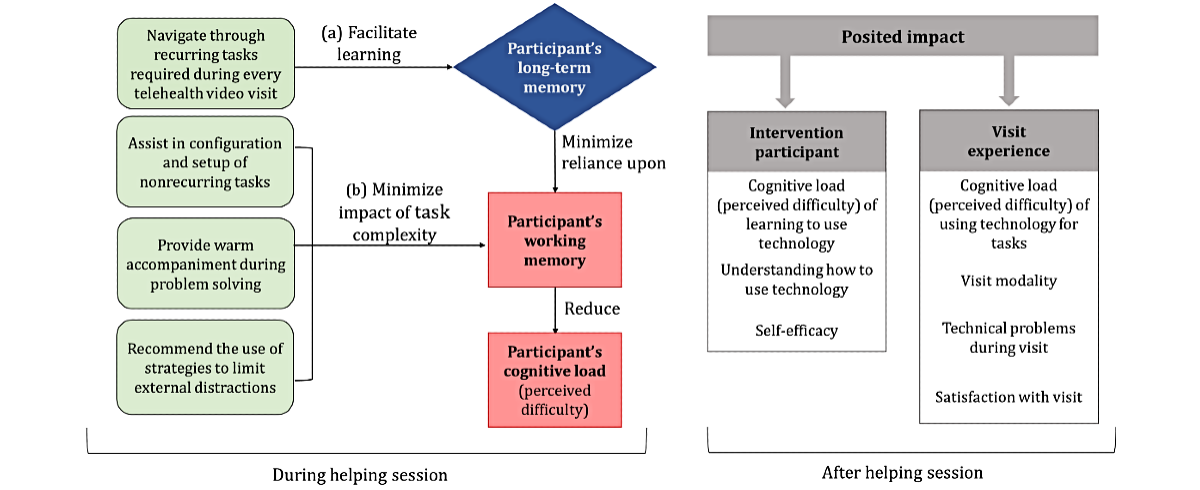
Theoretical foundations of intervention.

The different forms of memory referenced in the cognitive load literature [22-26] informed the intervention design (Figure 1). Working memory (or short-term memory) has a limited capacity and involves immediate, conscious activities of processing and organizing the information required to complete a task [25]. Long-term memory has infinite capacity and is where skills and knowledge are stored and drawn from to complete future tasks [25]. Successful task completion involves targeting cognitive load through the activation of long-term memory (Figure 1, blue diamond) and limiting the introduction of extraneous information that can tax working memory and cognitive load (Figure 1, red square boxes) [21,22,48,49].

As illustrated in Figure 1, the helping sessions targeted cognitive load reduction in 2 ways. First, having intermediaries navigate intervention participants through the recurring tasks required for a telehealth video visit facilitates learning (Figure 1, point a). Second, intermediaries can minimize the impact of task complexity by assisting in configuring nonrecurring tasks, providing warm accompaniment during problem-solving, and recommending strategies to limit environmental distractions (Figure 1, point b).

### Design of the Intervention

**Figure 2:**
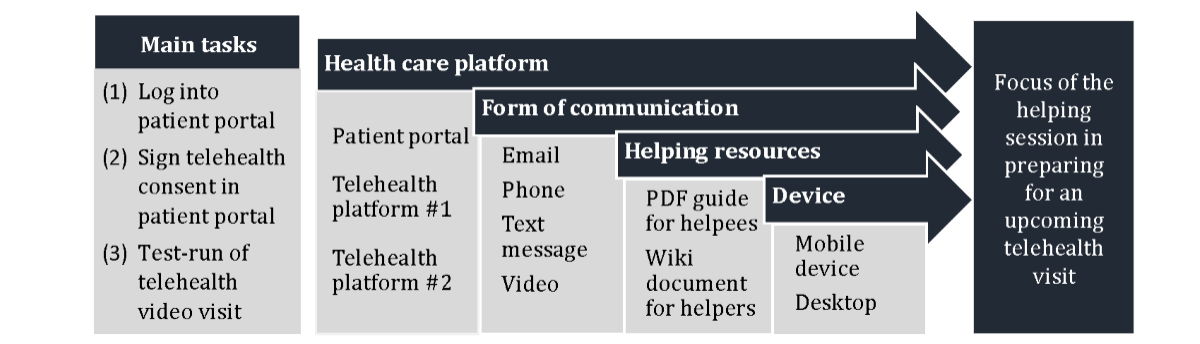
Design of intervention.

We designed the intervention to be a 10-30 minute *helping session* in which student intermediaries (ie, helpers) assisted patients with the 3 possible tasks. The direct guidance in task completion aligns with cognitive load theory’s emphasis on using modular “worked examples” in presenting novel information that can reduce cognitive load [50-52]. A PDF helping document was created in English and Spanish and designed to provide instructions for all 3 tasks. The document was sent to the intervention participants after the helping session, if they desired (Multimedia Appendix 1). The intervention was guided by each patient’s needs and interests, and thus we expected that patients would complete none, some, or all 3 tasks in a given helping session. Figure 2 illustrates how each helping session could have a different focus depending on the main tasks prioritized by the patient. For example, a helping session focused on logging into the patient portal might involve the helper talking through steps over the phone. At the same time, the intervention participants could receive emails on their mobile device to reset their password. By contrast, a test run on one of the telehealth platforms used by the clinic might involve the intervention participant receiving SMS text messages and turning on their video camera on their mobile device. At the same time, the helper accesses the Wiki document on their desktop.

Helpers were proficient in English or Spanish and paid graduate student research assistants trained as FQHC volunteers. Resources to help prepare helpers for the helping sessions included (1) a 1-hour orientation session, (2) a walkthrough of the intervention, (3) an intervention script (Multimedia Appendix 2), and (4) a Wiki document for problem-solving during the helping session (Multimedia Appendix 3). During the 2-week intervention, helpers held regular meetings to debrief on the process, troubleshoot through challenges, and share successful approaches applied during the helping sessions.

### Posited Impact of the Intervention

As the right side (green rounded boxes) of Figure 1 shows, we posited that the intervention could have the following impacts on the intervention participants by (1) reducing cognitive load by learning to use technology (video visit software and patient portals), (2) understanding how to use telehealth-related technology, and (3) enhancing self-efficacy in participating in video visits.

In addition, we posited that the intervention would impact participants’ visit experiences as follows: (1) reducing cognitive load by improving the performance of tasks related to video visits; (2) changing the visit modality from phone to video; (3) decreasing technical problems during video visits; and (4) improving satisfaction with video visits, including more willingness to recommend video visits to others.

### Participant Recruitment

Participants were patients from an FQHC in Metropolitan Detroit, who spoke either Spanish or English, and were over the age of 18 years. The helpers attempted to contact all patients scheduled for a phone or telehealth video visit during a 2-week period in August 2020. We established a limited time period because of the urgency of providing an alternative to face-to-face visits, while also recognizing that students would have more limited availability when their fall semester began. The FQHC partner did not provide advance notice to the patients before offering helping sessions. However, helpers called from a phone account that identified the organization’s name on the call display and the introductory script named the FQHC and the university as sponsors of the intervention. If helpers could not reach a patient by phone, a brief message was sent via voicemail or with the person who answered the call. Before the helping sessions, the intervention participants were asked to provide their oral consent and were mailed or emailed a copy of the informed consent documents.

### Ethics Approval

This study was reviewed and received ethics approval by the University of Michigan’s Institutional Review Board (#HUM00182442 and #HUM00152878).

### Data Collection

#### Process Evaluation

The multiple methods that we used for data collection are listed in Table 1. Helpers tracked recruitment effectiveness using Microsoft Excel to track the date, time, and method of communication when contacting potential participants. Moreover, helpers recorded structured observations [53] using a form during and immediately after the helping sessions with the patients (Multimedia Appendix 4), and they recorded structured reflection notes [54] on the intervention process at the end of each intervention day.

**Table 1:** Methods for data collection.

Methods	Description	Completed by	Evaluation measures
Intervention recruitment tracking	Microsoft Excel was used to track attempts to contact patients for the helping session. Information recorded included date and time of message and the results from a phone call (ie, voicemail, in-person message, no answer, busy, request for helper to call back, or refused to participate)	Helpers	Process evaluation
Structured observations	Completed during and after a helping session. Contained structured questions on visit modality and the locations of patients’ issues and open-ended questions on communication with patients and provided guidance	Helpers	Process evaluation
Reflection notes	Open-ended questions were completed at the end of each intervention day. These prompted helpers about their experiences, interactions and communication with patients, and their emergent techniques for completing intervention activities to reduce patient cognitive load	Helpers	Process evaluation
Telehealth experience survey	The survey was available in English and Spanish and contained validated measures on perceived difficulty as a measure of cognitive load, perceived usefulness, perceived ease of use, confidence in using telehealth, and satisfaction with visit. Designed to compare intervention and nonintervention participants	Intervention and nonintervention patient participants	Preliminary impact on intervention participant and visit experience
Telehealth experience survey: intervention-specific questions	A set of 4 questions were added at the end of the posttelehealth survey which asked helpees about their experiences with the technology helpers	Intervention patient participants	Preliminary impact on intervention participant
Semistructured interview experiences with patients who had helping sessions	Completed after a telehealth visit and asked patients about personal experiences with tasks, processes, and supporting technologies and their experiences with the helping sessions. Interviews were conducted in English or Spanish	Intervention patient participants	Process evaluation and preliminary impact on intervention participant
EHR^a^ data	Analyzed EHRs using manual chart review to assess whether the modality for the telehealth visit changed after the helping session and reasons provided for the modality change and to extract information on prior telehealth experience among intervention participants. Determined the number of portal users in providing details on the sociotechnical context	Research analyst	Preliminary impact on visit experience

^a^EHR: electronic health record.

#### Preliminary Impact of Helping Sessions

To assess the preliminary impact of the helping sessions and reach, we sent a survey link via an EHR-based text and portal “campaign” to (1) all adult patients at FQHC who spoke English or Spanish, (2) had consented to receive texts from the FQHC or had a patient portal, and (3) had completed a telehealth visit (phone or video) during or 4 weeks before and after the helping intervention pilot period. Accordingly, we contacted all eligible patients.

The survey contained validated measures on perceived usefulness [55,56], perceived ease of use [55,56], self-efficacy [57] in using telehealth, and perceived difficulty of tasks [58,59] and learning as measures of cognitive load. Satisfaction measures were drawn from the patient satisfaction survey at FQHCs [60] to facilitate comparisons within the FQHC. The full list of measures is presented in the *Results* section within the table on the preliminary impact.

Survey intervention participants were asked if they could be contacted for follow-up interviews. Those who indicated interest and intervention participants who did not complete the survey were contacted by phone or text for a 60-minute semistructured interview about their experiences with telehealth and the helping session. All interviews were recorded and transcribed, and Spanish transcripts were translated into English.

In addition, the EHR charts were manually reviewed to assess patients’ previous experiences with telephone or video visits and whether the final format of the scheduled telehealth visit had changed following the helping session. As applicable, we extracted the “reason” why patients opted for a phone visit from a drop-down list within the EHR visit note template accessed by all providers.

### Data Analysis

Three team members (GM, JJG, and LKB) analyzed survey data using descriptive statistics and inferential statistical tests, including the Wilcoxon rank-sum test, Pearson chi-square test, and Fisher exact test, using R software (R Foundation for Statistical Computing). Excel was used to categorize the data from the structured observations and chart reviews. One researcher (MA) used NVivo (QSR International) to code the data from the daily reflection notes, using both inductive and deductive coding (using codes informed by the cognitive load reduction literature) [22-25,48]. Qualitative analysis of the interviews, observations, and structured reflection notes guided the development of the process-related themes. Mixed analysis [61] consisted of 1 researcher (MA) writing analytic notes while comparing statistical tables on survey data, categorized observations and chart reviews, coded data, and interview transcripts. Consensus on process-related themes and pilot study recommendations involved an iterative process of refining analytic notes based on ongoing discussions with team members directly involved in the intervention. In addition, we drew from concepts in cognitive load theory [22-24] during our analysis to understand how intermediaries can address the cognitive load introduced through telehealth video visits. In particular, we applied the concepts of “elements” when considering the number of steps involved for each task, and “context-switching” when examining how the nonintegration of platforms introduced the need to juggle multiple windows on a single device.

## Results

### Reporting of Results

To inform future large-scale equity-focused interventions, we present the results on demographics for assessing recruitment and reach, in-depth reporting of our qualitative results for process delivery, and comparator data for evaluating the preliminary impact of the intervention [40,41,44]. In reporting demographics, we present recommendations for reporting results on health equity initiatives. Table 2 stratifies gender and age to provide further intersectional context as to who participated in the intervention [62]. Table 3 uses standardized categories [62] to provide a comparator for evaluating the intervention’s reach [41]. Both tables represent all participants’ data by reporting when there were missing data or preferences for not answering [63].

**Table 2:** Demographics of intervention participants (N=34).

Demographics			Female (n=24)		Male (n=8)		Preferred not to answer or missing (n=2)
**Age (years), n (%)**							
	18-25	2 (8)		0 (0)		0 (0)	
	26-35	1 (4)		2 (25)		0 (0)	
	36-45	10 (42)		1 (13)		0 (0)	
	46-55	7 (29)		3 (38)		0 (0)	
	56-65	4 (17)		1 (13)		0 (0)	
	66-75	0 (0)		1 (13)		0 (0)	
	>76	0 (0)		0 (0)		0 (0)	
	Preferred not to answer or missing	0 (0)		0 (0)		2 (100)	
**Race, n (%)**							
	Black	12 (50)		4 (50)		0 (0)	
	White	5 (21)		3 (38)		1 (50)	
	Other	7 (29)		0 (0)		1 (50)	
	Preferred not to answer or missing	0 (0)		1 (13)		0 (0)	
**Ethnicity, n (%)**							
	Hispanic	8 (33)		1 (13)		1 (50)	
	Non-Hispanic	16 (66)		6 (75)		1 (50)	
	Preferred not to answer or missing	0 (0)		1 (13)		0 (0)	
**Preferred language, n (%)**							
	English	17 (71)		7 (88)		2 (100)	
	Spanish	7 (29)		1 (13)		0 (0)	

**Table 3:** Demographic information and basic technological experience for survey participants (N=128).

Variable		Overall (N=128^a^)	No intervention (n=113^a^)	Intervention (n=15^a^)	*P* value^a,b^	
**Age (years; overall: n=121; no intervention: n=107; intervention: n=14)**						.90
	18-25, n (%)	14 (11.6)	12 (11.2)	2 (14.3)		
	26-35, n (%)	18 (14.9)	17 (15.9)	1 (7.1)		
	36-45, n (%)	34 (28.1)	29 (27.1)	5 (35.7)		
	46-55, n (%)	26 (21.5)	22 (20.5)	4 (28.6)		
	56-65, n (%)	20 (16.5)	19 (17.8)	1 (7.1)		
	66-75, n (%)	9 (7.4)	8 (7.5)	1 (7.1)		
	Preferred not to answer or missing, n	7	6	1		
**Education (overall: n=121; no intervention: n=106; intervention: n=15)**						.006
	≥High school, n (%)	99 (81.8)	91 (85.8)	8 (53.3)		
	<High school, n (%)	22 (18.2)	15 (14.2)	7 (46.7)		
	Preferred not to answer or missing, n	7	7	0		
Spanish-speaking survey participant, n (%)		23 (18)	18 (15.9)	5 (33.3)	.14	
**Internet experience**						.005
	Years, mean (SD)	14.1 (8.6)	14.9 (8.4)	8.4 (8.4)		
	Preferred not to answer or missing, n	17	16	1		
**Internet use frequency (overall: n=121; no intervention: n=107; intervention: n=14)**						.008
	Daily, n (%)	103 (85.2)	95 (88.8)	8 (57.1)		
	Several days a week, n (%)	8 (6.6)	5 (4.7)	3 (21.4)		
	Every few weeks to never, n (%)	10 (8.3)	7 (6.5)	3 (21.4)		
	Preferred not to answer or missing, n	7	6	1		
**How often do you need to have someone help you when you read written material from your physician or pharmacy? (overall: n=122; no intervention: n=107; intervention: n=15)**						.27
	Never, n (%)	102 (83.6)	91 (85)	11 (73.3)		
	Not never, n (%)	20 (16.4)	16 (14.9)	4 (26.7)		
	Preferred not to answer or missing, n	6	6	0		
**Access to any technology (overall: n=121; no intervention: n=106; intervention: n=15)**						.03
	>1, n (%)	81 (66.9)	74 (69.8)	7 (46.7)		
	1, n (%)	39 (32.2)	32 (30.2)	7 (46.7)		
	0, n (%)	1 (0.8)	0 (0)	1 (6.7)		
	Preferred not to answer or missing, n	7	7	0		
**Access to desktop (overall: n=114; no intervention: n=99; intervention: n=15)**						.54
	No, n (%)	69 (60.5)	61 (62.2)	8 (53.3)		
	Yes, n (%)	45 (39.5)	38 (38.8)	7 (46.7)		
	Preferred not to answer or missing, n	14	15	0		
**Access to laptop (overall: n=120; no intervention: n=105; intervention: n=15)**						.26
	No, n (%)	48 (40)	40 (38.1)	8 (53.3)		
	Yes, n (%)	72 (60)	65 (61.9)	7 (46.7)		
	Preferred not to answer or missing, n	8	8	0		
**Access to cell phone (overall: n=119; no intervention: n=104; intervention: n=15)**						.13
	No, n (%)	1 (0.8)	0 (0)	1 (6.7)		
	Yes, n (%)	118 (99.1)	104 (100)	14 (93.3)		
	Preferred not to answer or missing, n	9	9	0		
**Access to tablet (overall: n=115; no intervention: n=100; intervention: n=15)**						.23
	No, n (%)	68 (59.1)	57 (57)	11 (73.3)		
	Yes, n (%)	47 (40.9)	43 (43)	4 (26.7)		
	Preferred not to answer or missing, n	13	13	0		
**Access to book device (overall: n=114; no intervention: n=100; intervention: n=14)**						.44
	No, n (%)	97 (85.1)	86 (86)	11 (78.6)		
	Yes, n (%)	17 (14.9)	14 (14)	3 (21.4)		
	Preferred not to answer or missing, n	14	13	1		
**Access to game console (overall: n=114; no intervention: n=101; intervention: n=13)**						.99
	No, n (%)	111 (97.4)	98 (97)	13 (100)		
	Yes, n (%)	3 (2.6)	3 (3)	0 (0)		
	Preferred not to answer or missing, n	14	12	2		

^a^Pearson chi-square test; Wilcoxon rank-sum test; Fisher exact test.

^b^Bonferroni correction not used. Sample size discrepancy considered within tests.

### Characteristics of Participants

#### Intervention Participants

As detailed in Table 2, the study intervention participants (N=34) were an average of 44 years of age, 71% (24/34) were women, 24% (8/34) were men, and none were identified as nonbinary. Regarding race and ethnicity, 47% (16/34) identified as Black, 24% (8/34) as White, and 27% (9/34) as Hispanics or Latinx. For the primary language, 24% (8/34) of participants spoke Spanish, and 79% (24/34) spoke English.

#### Survey Participants (Including Intervention Participants and Nonintervention Participants)

Table 3 presents the demographics of the survey participants (N=128). The mean age was 44.4 (13.7 SD) years for nonintervention participants and 43.9 (12.4 SD) years for intervention participants, and no participants were over the age of 75. A lower percentage (18/113, 15.9%) of nonintervention participants completed the survey in Spanish than the intervention (5/15, 33%) participants.

#### Interviewed Intervention Participants

The 3 participants who completed the intervention, survey, and interview had a mean age of 43 years and identified as a Black non-Hispanic man, a White Hispanic woman, and a White non-Hispanic man.

### Process Evaluation

#### Feasibility of Recruitment Methods for Intervention

**Figure 3:**
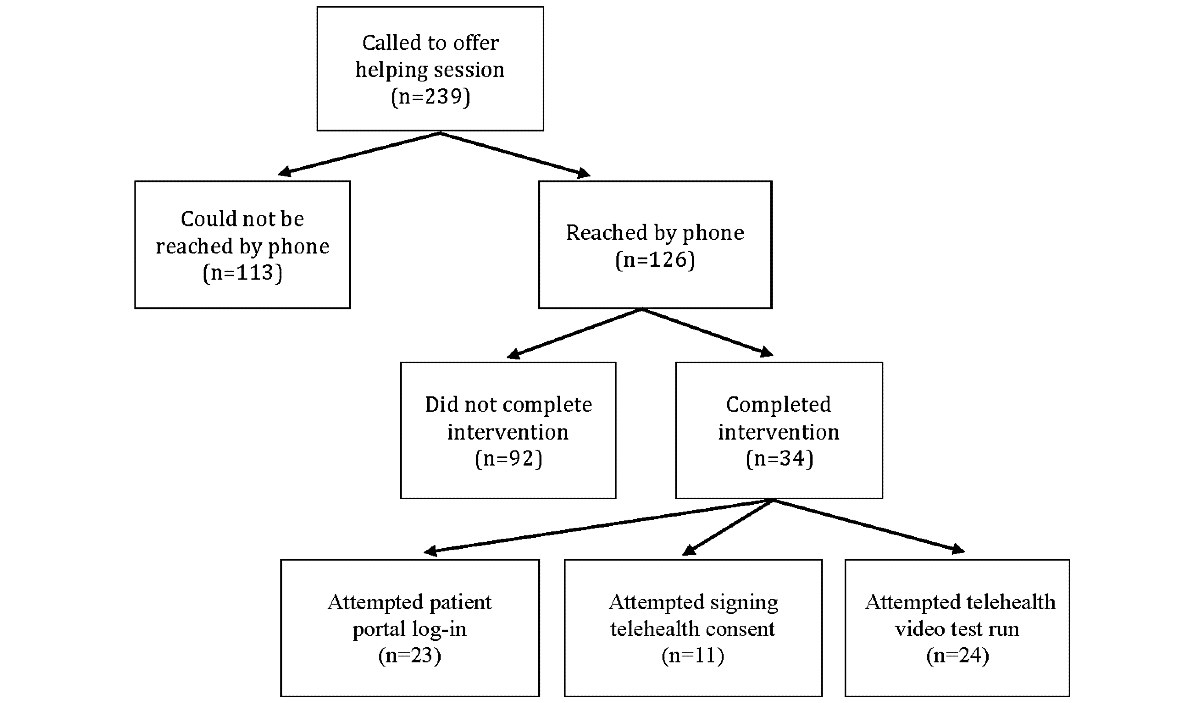
Recruitment for the intervention.

As shown in Figure 3, close to half (113/239, 47.3%) of the intervention-eligible participants could not be reached by phone, 52.7% (126/239) answered the phone, and 14.2% (34/239) participated in the intervention. Table 4 illustrates that most of the intervention participants (76.4%) were called within a day of their appointment, and none of the patients who were called a week or more before their appointment participated.

We found that intervention recruitment was more successful when we could reach people directly over the phone versus via voicemail (Table 4). When we reached people directly on the first phone call, 16 people immediately participated in the helping session and 49 asked for a callback. Of those who asked for a callback, 15 participated in the study. In contrast, none of the 77 eligible patients we left a voicemail with participated.

Multimedia Appendix 5 details why people who were reached directly by phone did not participate in the study. Many people (n=27) stated that they were either not interested, had the required skills, or were busy, whereas 7 people stated a lack of comfort with technology, access to technology, or skills required for a telehealth video visit. In addition, 2 people stated that they were distrustful of calls. However, having helpers representing familiar organizations was important in gaining a patient’s trust in finding the intervention. This was illustrated by a reflection note: when the person answered the phone they were “intimidating and [were] concerned this was a spam call—upon hearing I was connected to [the clinic] and university they were eager to give the phone to [their] son to communicate with me.”

**Table 4:** Feasibility of recruitment for intervention: timing and tracking of calls.

			Participated in intervention (n=34)		Did not participate in intervention (n=205)
**Number of days between intervention and telehealth visit, n (%)**					
	0 (same day)	12 (35.3)		35 (17.1)	
	1	14 (41.2)		80 (39)	
	2 to 3	3 (8.8)		31 (15.1)	
	4 to 6	5 (14.7)^a^		25 (12.2)	
	More than 6 days	0 (0)		34 (16.6)	
**Phone was answered on first call, n (%)**					
	Immediately participated in helping session	16 (47.1)		N/A^b^	
	Gave an immediate response not to participate	N/A		43 (21)	
	Requested a call back	15 (44.1)		34 (16.6)	
	Message was left with a person	2 (5.9)		8 (3.9)	
**Phone was not answered on first call, n (%)**					
	Voicemail	0 (0)		77 (37.6)	
	No answer	1 (2.9)		29 (14.1)	
	Missing	0 (0)		14 (6.8)	

^a^One visit was canceled after completing the helping session.

^b^N/A: not applicable.

#### Reach of the Intervention: Survey Results

**Figure 4:**
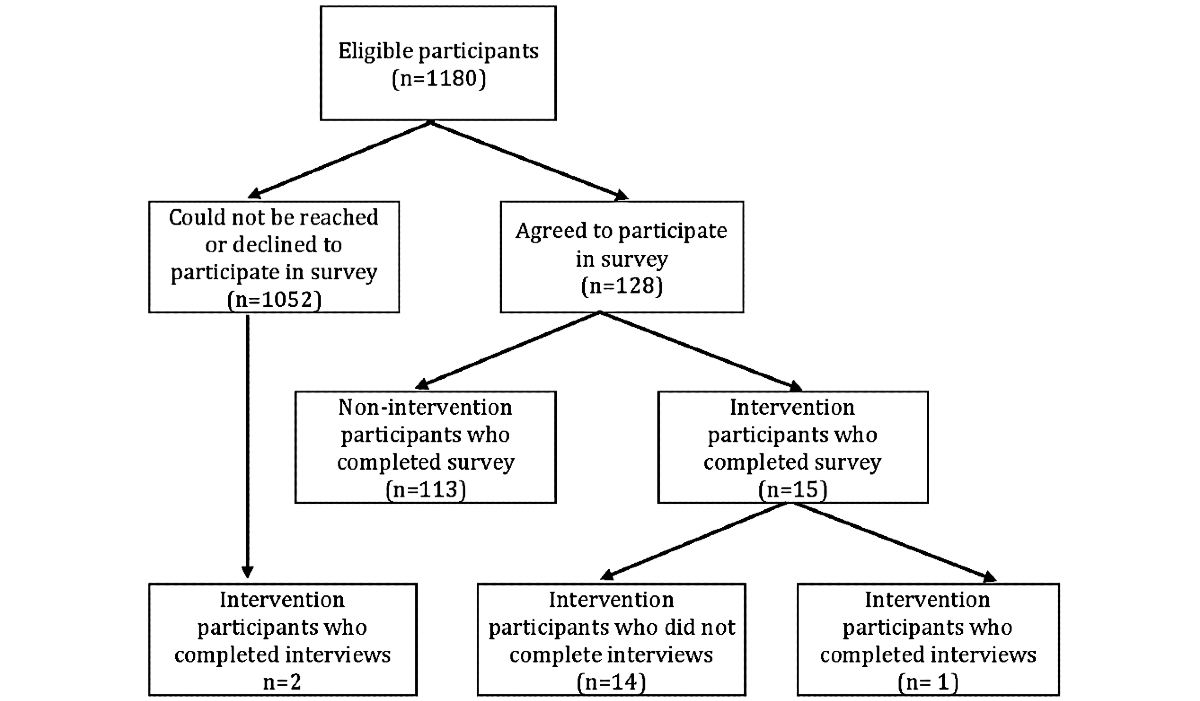
Recruitment for the telehealth experience survey and interviews.

Figure 4 details that of the 1180 patients invited to participate in the survey, 113 nonintervention participants and 15 intervention participants completed the survey (10.8% overall survey response rate; and 44% of participants who had the intervention). The survey results were used to evaluate reach of the intervention by comparing demographics and telehealth experiences between intervention and nonintervention participants. Table 3 shows that we successfully recruited patients at FQHC to the intervention who would likely benefit from the helping sessions. Intervention survey participants were more likely to have less than a high school education (15/106, 14.2% vs 7/15, 47%), fewer years of internet experience (8.4 years vs 14.9 years) and report a lower percentage of daily internet and email use (8/14, 57% vs 95/107, 88.8%) when compared with nonintervention participants. Though we found no significant association, compared with nonintervention participants (16/107, 14.9%) more intervention survey participants (4/15, 27%) needed help when reading written material from their physician or pharmacy, which indicates lower health literacy [64].

Table 3 also shows that the intervention participants were equally likely to have access to technological devices. However, nonintervention participants (74/106, 69.8%) were more likely to have access to more than 1 device than intervention participants (7/15, 47%). The sole survey participant who did not have access to any technology (although they expected to have access to a smartphone shortly) also participated in the intervention.

#### Task Attempts and Completion

The helping sessions ranged from 5 to 65 minutes, with an average time of 34 minutes. In the 8 sessions that were 50 minutes or longer, 7 resulted in the successful completion of all 3 tasks. Table 5 shows the tasks that the helpers attempted and completed during the 34 helping sessions. The 8 unresolved patient portal issues were due to the intervention participants not being able to locate the sign-in link for the patient portal invited (n=3), having difficulties in resetting the password (n=2), electing to finish patient portal registration later (n=1), loss of interest (n=1), and different page views between the helper and the participant (n=1).

During the helping session, 11 participants attempted to sign the telehealth consent in the patient portal, 5 of whom were successful (Table 5). Helpers discovered early in the delivery of the intervention that once patients signed the telehealth consent form, it was no longer visible on the patient portal. This design made it challenging for helpers to fully evaluate why 6 intervention participants could not complete the telehealth consent task during the session. Intervention participants may have been in the wrong section of the patient portal or they may have already provided consent during a previous video visit. After the sessions, we determined that 4 of these intervention participants had previously had a telehealth visit, and thus, it was probable that they had previously signed the telehealth consent form.

During the helping session, 24 intervention participants attempted a telehealth video test run (Table 5). A telehealth video test run was not attempted in 4 of the helping sessions, as the focus was on setting up the patient portal to review telehealth consent. Additional reasons for not completing the telehealth video test run included the intervention participants having prior telehealth video visit experience and not wanting a refresher (n=4), lack of a device to support video visits (n=1), or lack of interest in the test run (n=1). The 8 unsuccessful telehealth video test runs were due to poor sound quality (n=3), inability to connect to the telehealth link (n=4), or patient not being fully interested in the support provided (n=1). Helpers resolved issues during the 16 successful telehealth video test runs by asking intervention participants to enter device settings to increase volume, open a different browser, or close external programs to maximize bandwidth.

**Table 5:** Tasks attempted and completed by participants during helping session (N=34).

Task		Number of participants, n (%)	Successful on task, n (%)	Not successful on task, n (%)
**Task 1: Log into patient portal**				
	Attempted to resolve patient portal issue	23 (68)	15 (65)^a^	8 (35)^a^
	Did not attempt to resolve a patient portal issue	11 (32)	N/A^b^	N/A
**Task 2: sign telehealth consent**				
	Attempted to sign telehealth consent	11 (32)	5 (45)^c^	6 (55)^c^
	Did not attempt to sign telehealth consent	23 (68)	N/A	N/A
**Task 3: conduct telehealth video test run**				
	Attempted telehealth video test run	24 (71)	16 (67)^d^	8 (33)^d^
	Did not attempt telehealth video test run	10 (29)	N/A	N/A

^a^n=23.

^b^N/A: not applicable.

^c^n=11.

^d^n=24.

#### Task “Elements” and “Context-Switching”

**Figure 5:**
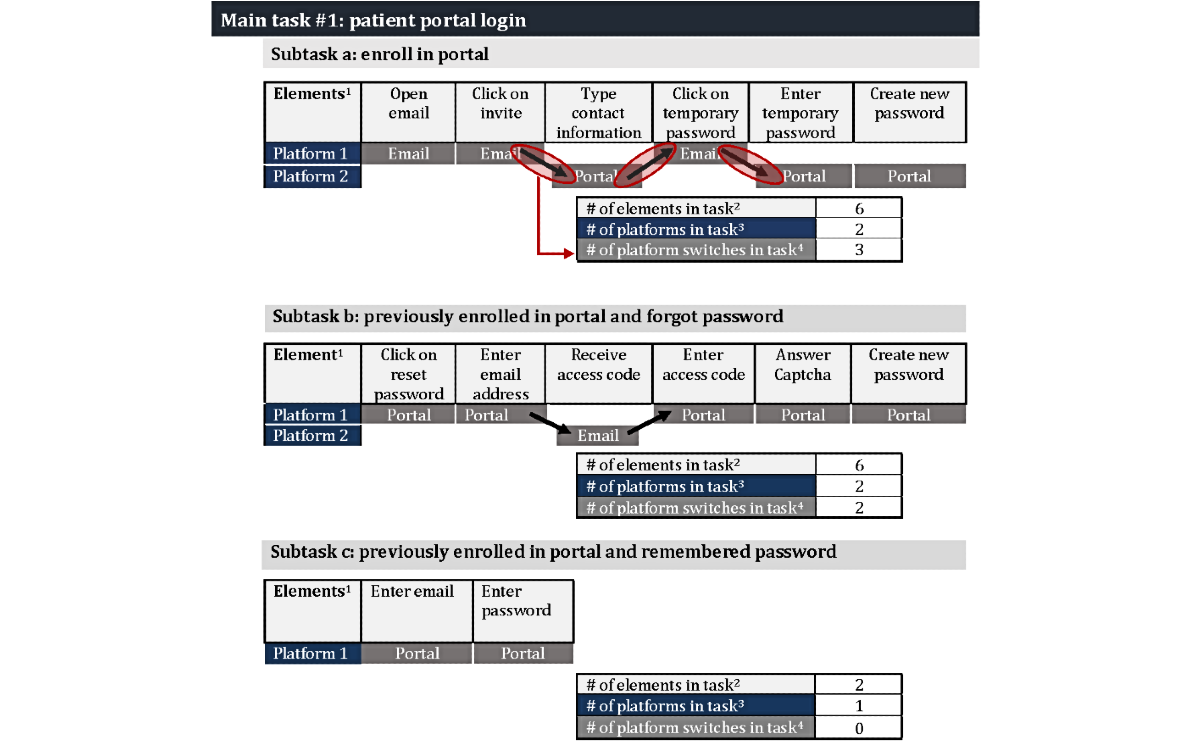
Number of elements and platform switches required for patient portal log-in tasks. ^1^The verbs used for each element illustrates the variety of actions required for each three tasks; ^2^number of boxes in the element row; ^3^number of rows of shaded boxes in the platform; and ^4^number of arrows; each arrow indicates a platform switch; dual arrows indicate there are 2 possible pathway.

**Figure 6:**
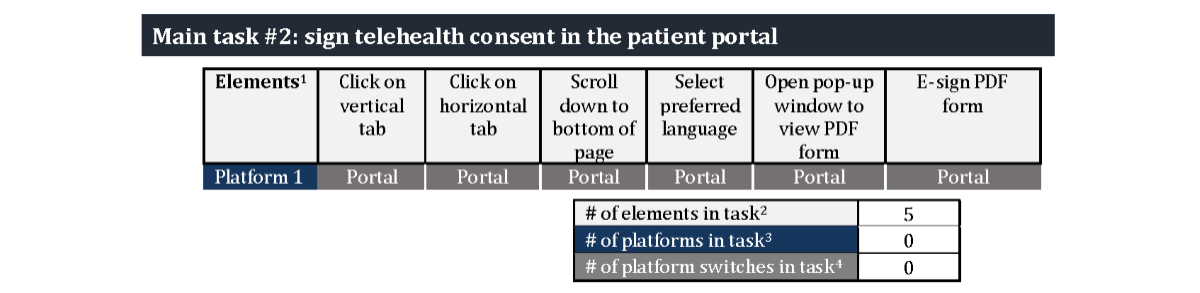
Number of elements and platform switches required for telehealth consent tasks. ^1^The verbs used for each element illustrates the variety of actions required for each three tasks; ^2^number of boxes in the element row; ^3^number of rows of shaded boxes in the platform; and ^4^number of arrows; each arrow indicates a platform switch; dual arrows indicate there are 2 possible pathway.

**Figure 7:**
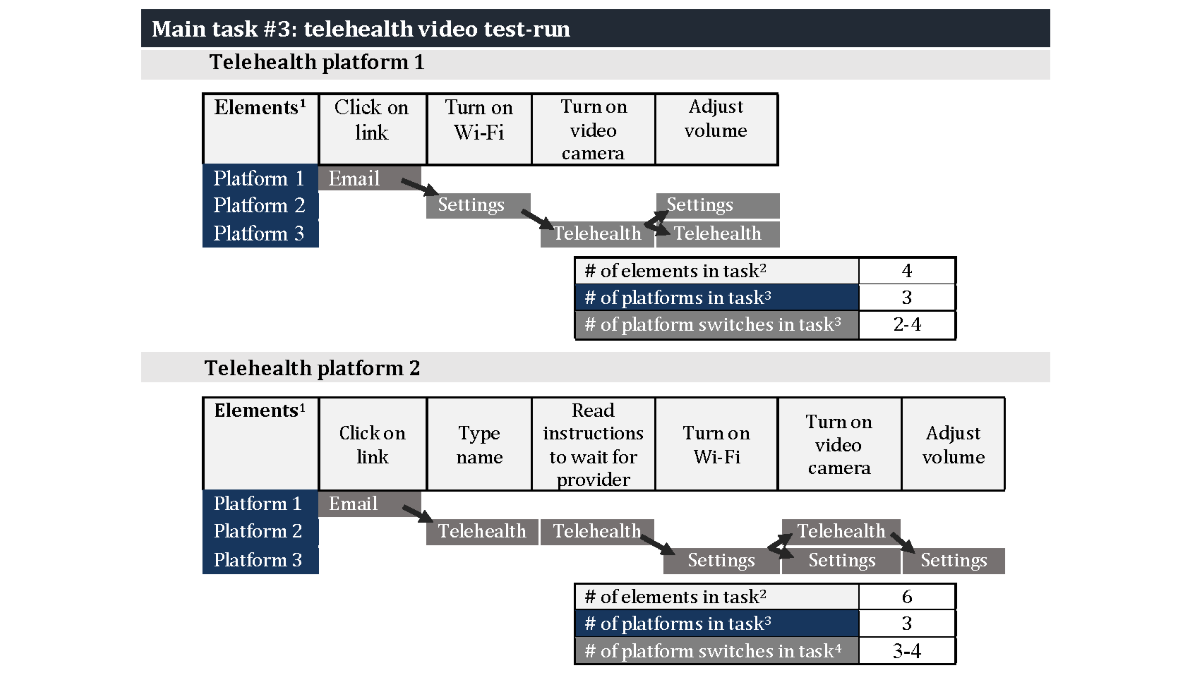
Number of elements and platform switches required for telehealth tasks. ^1^The verbs used for each element illustrates the variety of actions required for each three tasks; ^2^number of boxes in the element row; ^3^number of rows of shaded boxes in the platform; and ^4^number of arrows; each arrow indicates a platform switch; dual arrows indicate there are 2 possible pathway.

Elements within the 3 tasks introduced distinct language, novel processes, and context-switching between platforms, which could possibly influence task completion. Figures 5-7 illustrate the potential impact by detailing the number of elements, variety of elements, and context-switching involved for the three tasks: (1) logging into the patient portal (6 elements and 3 platform switches), (2) signing the telehealth consent (6 elements and 0 platform switches), and (3) conducting a telehealth video visit (6 elements and 3-4 platform switches). The verbs used to describe the elements (eg, open, click, and enter) in Figures 5-7 illustrate their variety, and thus, a possible introduction to novel processes.

In addition, Figure 5 shows how the different pathways for the patient portal log-in task depended on the intervention participants’ previous experiences with the portal and their familiarity with the required elements. Some participants who needed to reset their portal password also had limited patient portal experience and were less familiar with how to use their email. These descriptions from the helpers further exemplify the difficulties encountered when using email to reset the patient portal passwords:

They kept telling me their email handle was email.com instead of gmail.com. I found out it was Gmail when I asked who provided the email service she uses.

He entered his email and confirmed it multiple times, but he was not able to see an email from [FQHC] in his email. He wasn’t very familiar with the Gmail app that was on his phone.

The sessions that focused on completing the task of resetting their password and logging into the patient portal often involved the introduction of a new set of elements and novel processes for signing telehealth consent in the patient portal (Figure 6). In contrast, the 5 sessions that did not involve accessing the patient portal allowed participants to avoid these additional elements and directly attempting a telehealth video test run.

The providers had different preferences for using the 2 telehealth video platforms, both of which lacked patient portal integration. One of the telehealth video platforms offers a simplified process involving a single step of clicking on a link sent via a text message. However, during the telehealth video test runs, the intervention participants had to switch context between different areas of their phones because they were required to navigate between the telehealth platform and device settings for sound and video adjustments (Figure 7).

#### Task Complexity and Participants’ Sociotechnical Context

Helpers had a limited ability to minimize external factors that could impact a participant’s cognitive load. Although helpers encouraged intervention participants to find a quiet physical environment for the sessions, this was not always possible because of background noise from others in the household. In addition, household members may have been at home during the early days of the COVID-19 pandemic, which could have potentially introduced distractions during telehealth video test runs. A helper noted how external factors impacted the session:

[The participant was] unable to focus attention on the task at hand. I had to repeat questions and processes multiple times, patient repeatedly spoke to other people.

The intervention participants experienced internet access disruptions during the helping sessions, which may have increased during the high service times of the day. In addition, they needed to use their data plans for the telehealth video test runs. Most intervention participants used mobile phones as their only devices. Thus, the required tasks involved using a small screen to switch between email, SMS text messages, patient portal, and telehealth platform. Often, these mobile devices were older and no longer offered current technical documentation for troubleshooting. The screen was damaged in at least one case. One helper noted these varied issues in the structured observation form:

[The] phone was cracked and hard to use. Was trying to get a new phone to be able to do visits better. Doesn’t have Wi-Fi so was planning on using data for the [video visit] call...Call disconnected 2-3 times. Had bad service.

#### Helpers’ Emergent Approaches to Reducing Cognitive Load

##### Overview

As the intervention progressed, helpers demonstrated that their ability to assist patients was not from knowing every single technical solution detailed in a Wiki document or manual. Rather, they developed tactics to target complex and uncertain technical pathways. Themes based on analyses of helper-completed forms and reflection notes demonstrated how the 4 helping session activities expanded into the techniques of *providing step-by-step guidance for configuring and learning*, *building rapport to establish confidence while problem-solving* and *being on the same page to counter informational distractions*. These 3 themes are further detailed below. Multimedia Appendix 6 provides examples from our data to further illustrate how and when to use these techniques to target cognitive load.

##### Theme 1: Providing “Step-by-Step” Guidance for Configuring and Learning

The step-by-step guidance offered during the helping sessions supported configuration and learning. During an interview, an intervention participant noted the importance of this approach when they said:

[The helper’s] step by step [guidance on] how to do the link...how to fill out the questionnaire...and where to go and where to send the link and everything, and how the process went...and little by little she explained it to me.

Helpers demonstrated using a stepwise approach during configuration and setup by intentionally pausing so that they could confirm with intervention participants that they were both at the same stage. One helper also demonstrated how confirmation of each step helped determine why something may not be working for the intervention participant:

I walked him through the steps again to connect to [the video platform] and checked in after each step. For example, did he receive the link via text?...click on the link?...Eventually we figured out the blockage was that his phone said “another app is accessing the camera and or microphone.”...[We] disconnect[ed] the phone call and see if that allowed him to get onto [the video platform] and it did.

A “step-by-step” approach was also helpful in guiding participants through context switches between platforms and device configurations. One helper illustrated that storing information from a previous step while the participant moved to the next step could be helpful when switching between platforms:

I told him that if he wanted help remembering the security code to get into his account, he could tell it to me, and I could repeat it back to him when he went to plug the number in for verification. This proved to be helpful as he forgot the number when we left the text message.

##### Theme 2: “Building Rapport” to Establish Confidence While Problem-Solving

Given the warm accompaniment design of the intervention, helpers accorded particular attention to building rapport with the intervention participants. Helpers noted that many intervention participants were interested in obtaining more information about their upcoming telehealth visits, whereas others demonstrated negative affect. For example, helpers described intervention participants as “interested but somber,” or initially “hesitant,” “cautious” or “distrusting.” In such situations, building a rapport is of even greater importance.

Helpers observed that rapport was often built during impromptu moments, which allowed for more natural conversation, shared cultural connections, and a shift to the participant’s priorities. Examples included when a helper “flubbed the lines” with the participant, they shared “a good laugh,” and from that point on the session “felt more conversational.” During another session that began in English, the participant picked up cues from the helper that they were both Spanish-speaking, and they switched to speaking “*Spanglish,*” making the session more relaxed. Another session demonstrated added value when the helper dedicated time to the participant’s request for instructions on sending a message to their health care provider in the patient portal.

The shared emotions of the intervention participants and helpers completing novel tasks illustrated another source for building rapport. In the initial days of the intervention, helpers wrote about being “frustrated” and felt like they were “stabbing in the dark” when trying to obtain the signed telehealth consent form. However, helpers began noting how the intervention participants reciprocated patience and the subsequent sense of accomplishment in working together through this shared problem. Helpers saw that the helping session had not only “built [participants’] confidence in knowing what to expect” but that the sessions were also “a confidence builder for me [the helper].” Helpers also shared moments of happiness when completing a task with participants who initially doubted their abilities. This was demonstrated by a helper’s reflection note:

Finally SUCCESS!! We were both so happy.” During a follow-up interview, an intervention participant demonstrated a helper’s success in building rapport when they noted that it was “...as if she was a friend...she was calming [and] I always felt comfortable”

##### Theme 3: “Being on the Same Page” to Counter Informational Distractions

Helpers’ efforts to “being on the same page” was both a literal and figurative endeavor in being able to see the same view on the screen as patients and in developing a shared understanding with them. During the intervention, the importance of “being on the same page” became evident when helpers discovered that they had a different view of the patient portal than the intervention participants. After encountering this issue early in the intervention, helpers got on the same page by learning to switch to a mobile phone view during the intervention.

In addition, “being on the same page” involved confirming the terms and words used during the helping sessions. A helper related how they, “talk[ed] back [to the patient to]...make sure we meant the same thing.” In contrast, the introduction of unfamiliar technical terms demonstrated that unfamiliar words could disrupt processes for “being on the same page.” The interviews offered an example of this when a participant did not know how to respond to questions about using “the patient portal” and responded with confusion “what?” and was then able to answer the question when asked whether they use “the online web site...for test results and upcoming visits.” Notably, encountering novel terms can be a potential source of complexity that can increase the cognitive load [22].

#### “Warm Accompaniment” Approach

Postintervention data collection focused on gathering feedback about the helping session through surveys and interviews. As detailed in Figure 4, our team had limited success in recruiting intervention participants for these stages: out of the 34 intervention participants, 15 participated in the survey and 3 completed interviews. Only 6 intervention participants responded to the survey questions regarding their experiences with the helping sessions. Survey questions probed the extent to which the helpers succeeded in implementing our “warm accompaniment” strategy using a Likert scale response of agreement (1=Strongly Disagree to 5=Strongly Agree). Intervention participants indicated that, on average, they agreed that “The technology assistant cared about me as a person” (mean 4.3, SD 0.8) and strongly agreed that the helper “really tried to help me” (mean 4.5, SD 0.8).

### Preliminary Impact of the Intervention

Our limited survey responses and interviews with intervention participants affected our ability to assess the preliminary impact of the intervention on the participants. However, given that pilot studies are exploratory, we present the preliminary findings below and in Table 6 to inform the design of future large-scale equity-focused studies [41].

**Table 6:** Evaluation of the preliminary impact of the intervention (Telehealth Experience Survey Results; N=128).

				Overall (n=61)		No intervention (n=54)		Intervention (n=7)		*P* value^a^
**Preliminary impact on the intervention participants: cognitive load when learning to use technologies related to video visits (1: very difficult; 5: very easy)**										
	**Learn how to use the video visit software**									
		Value, mean (SD)	4.4 (0.8)		4.5 (0.7)		3.6 (1.3)		.02	
		Preferred not to answer, n	1		1		0			
	**Learn how to use the patient portal to get my laboratory orders, imaging referrals, test results and follow-up instructions**									
		Value, mean (SD)	3.8 (1.1)		3.8 (1.1)		3.3 (1.1)		.24	
		Preferred not to answer, n	3		3		0			
**Preliminary impact on the intervention participants: self-efficacy (certainty that I can connect to a video; 0%-100%)**										
	**On my current device**									
		Value, mean (SD)	92.6 (20.4)		94.1 (16.0)		79.6 (44.5)		.27	
		Preferred not to answer, n	12		10		2			
	**On a new device**									
		Value, mean (SD)	85.2 (26.0)		87.1 (23.2)		73.0 (40.6)		.35	
		Preferred not to answer, n	15		14		1			
	**When someone else is helping me**									
		Value, mean (SD)	91.3 (21.6)		92.4 (17.9)		83.3 (40.8)		.86	
		Preferred not to answer, n	14		13		1			
	**When no one is helping me**									
		Value, mean (SD)	90.0 (24.3)		89.3 (25.5)		96.0 (8.9)		.66	
		Preferred not to answer, n	13		11		2			
	**Self-efficacy measure average**									
		Value, mean (SD)	88.6 (21.8)		90.3 (18.5)		75.7 (39.1)		.26	
		Preferred not to answer, n	10		9		1			
**Preliminary impact on visit experience: cognitive load when performing tasks related to video visits (1: very difficult; 5: very easy)**										
	**Use the software to have a visit with my health care provider**									
		Value, mean (SD)	4.4 (0.8)		4.5 (0.7)		3.7 (1.3)		.047	
		Preferred not to answer, n	0		0		0			
	**Use the patient portal to get my laboratory orders, imaging referrals, test results and follow-up instructions**									
		Value, mean (SD)	3.7 (1.2)		3.8 (1.2)		3.0 (1.2)		.07	
		Preferred not to answer, n	3		3		0			
**Preliminary impact on visit experience: satisfaction (1: very dissatisfied or very unlikely; 5: very satisfied or very likely)**										
	**How satisfied were you with your recent phone visit experience?**									
		Value, mean (SD)	4.3 (0.9)		4.3 (0.9)		4.6 (0.5)		.30	
		Preferred not to answer, n	61		54		7			
	**How likely would you be to tell your friends and family to use phone visits?**									
		Value, mean (SD)	4.2 (0.9)		4.2 (0.9)		4.4 (0.5)		.67	
		Preferred not to answer, n	62		54		8			
	**How satisfied were you with your recent video visit experience?**									
		Value, mean (SD)	4.4 (1.0)		4.5 (0.9)		3.3 (1.2)		.002	
		Preferred not to answer, n	70		61		9			
	**How likely would you be to tell your friends and family to use video visits? mean (SD)**									
		Value, mean (SD)	4.5 (0.6)		4.6 (0.6)		4.3 (0.8)		.27	

^a^Wilcoxon rank-sum test.

#### Impact on Intervention Participant

##### Cognitive Load When Learning to Use Technology

In terms of cognitive load and learning, Table 6 shows that, on average, participants found that using the patient portal software was more difficult to learn than the video visit software. In addition, intervention participants were more likely to find it more difficult to learn to use the video visit software than were nonintervention participants. However, there was no significant difference in the difficulty in learning how to use the patient portal.

##### Understanding How to Use Technology

From the 6 survey responses about the helper enhancing *understanding* of using technology, we found agreement (mean 4.3, SD 1.2) on *understanding* how to use the video visit software, and weak agreement (mean 3.7, SD 1.5) on *understanding* how to use the patient portal.

##### Self-efficacy

On average, participants indicated high certainty of being able to connect to video visits in a range of scenarios, although scores were lower on the hypothesized new device (Table 6). We found no significant differences between the groups for self-efficacy (Table 6); however, intervention patients had, on average, 14.6% fewer percentage points in their ability to connect to a video visit in any scenario proposed from the self-efficacy questions.

#### Impact on Visit Experience

##### Cognitive Load When Performing Tasks Related to Video Visits

As for our measure related to cognitive load when performing tasks (Table 6), we found no significant differences regarding perceived difficulty in using the video visit software to visit participants’ health care providers. We also found no significant differences between the 2 groups in terms of how difficult it was to use the patient portal. Notably, both participant groups found the patient portal to be more difficult to use than the telehealth platform.

##### Visit Modality

Of the 34 telehealth visits, before the helping session 74% (n=25) were scheduled as video visits and 26% (n=9) were scheduled as phone visits. The percentage of visit modalities recorded after the telehealth visit was 53% (18/34) via video and 47% (16/34) via phone. Overall, the EHR notes had the following reasons for the 16 phone visits: no video available at the time of the visit (n=6), technical difficulties (n=4), patient refusal or preference (n=4), and no reason given (n=2).

Following the helping session, 30% (13/34) of the 3 intervention participants’ visit modalities had changed. Of the 9 intervention participants initially scheduled for a phone visit, 3 (33%) switched to video for their telehealth visit. Among the 3 intervention participants, 2 (66%) had prior phone visits, 1 (33%) had a prior video visit, and 1 (33%) had a successful video visit test run during a helping session. Of the 25 intervention participants initially scheduled for a video visit, 10 (40%) ultimately had a phone visit. Of those who switched from video to phone, 5 had prior phone visits and 1 had a prior video visit. Of the 10 intervention participants initially scheduled for video visits and switched to phone, 7 had conducted a successful telehealth video test run during the helping session. For those who had successful video test runs but still switched to the phone, the EHR notes stated that the phone was due to video not being available (n=2), technical difficulties (n=2), patient refusal or preference (n=1), and no reason given (n=2). There was 1 individual who did not have a device to support video visits at the time of the session, but the helper successfully walked the participant through the process to prepare them for when their new device arrived.

In addition, 62% (21/34) of patients did not experience a change in visit modality, of whom 15 had video telehealth visits and 6 had phone telehealth visits. Of these 15 video telehealth visits, 6 participants had prior phone visits and 6 participants had prior video visits. In addition, 6 participants who had a telehealth video visit completed a video visit test run during a helping session, of whom 4 had never had a prior video visit. Multimedia Appendix 7 provides a summary of phone and video telehealth visits by nonintervention and intervention survey participants.

##### Technical Problems During Visits

Although there was no significant relationship, overall, the survey participants reported more problems during video visits (9/66, 14%) than during phone visits (3/72, 4%). We found that most of the video visit problems reported in the survey occurred during the visit and were concerned with audiovisual quality (Multimedia Appendix 7).

##### Satisfaction

As for satisfaction (Table 6), the intervention survey participants were significantly less satisfied with their video visit experience compared with the nonintervention participants. However, there were no significant differences between the groups in terms of satisfaction with the phone visits. In addition, we found no significant difference between the 2 groups regarding the likelihood of telling friends or family members to use phone visits or video visits.

## Discussion

### Principal Findings

Our pilot study evaluated the reach delivery process and the preliminary impact of a telehealth intervention for preparing patients at FQHC for an upcoming telehealth visit. Despite the limited sample size, our study found that people were likely to benefit from the intervention. Of the 3 tasks, most helping sessions focused on logging into the patient portal and conducting a telehealth test run, of which approximately two-thirds resulted in successful task completion. We found less success in helping sessions that involved signing the telehealth consent form; however, this may have been because of intervention participants previously signing the form, and thus was no longer visible.

Helpers’ emergent techniques in targeting the cognitive load experienced by intervention participants included: *providing step-by-step guidance for configuring and learning*, *building rapport to establish confidence while problem-solving* and *being on the same page to counter informational distractions*. The intervention participants demonstrated the importance of the “warm accompaniment” design when they agreed with items concerning helpers’ caring when offering help. As for preliminary impact, intervention participants reported an increased understanding of technology, and intervention participants rated greater difficulty in learning the video visit software than other patients at FQHC. However, there was no significant difference in the self-rated difficulty of learning to use the portal. When considering visit experience, there was no difference in the cognitive load of using technology, and we did not find any impact on the visit modality or technical problems during the visit. Intervention survey participants were more likely to be less satisfied with their video visits than were nonintervention participants.

### Comparison With Prior Work

#### Recruitment Feasibility: Method of Communication

The success of our equity-focused study was that we reached people with less technological experience, who are often underrepresented in technological intervention studies [47]. Compared with nonintervention participants, intervention participants had less formal education, fewer years of internet experience, access to fewer technological devices, and used technology less often. Of the 90 people who refused to participate, only 9 indicated technical reasons as barriers to participation, and 28 indicated being busy or lacking need or interest. These recruitment findings suggest that those who reached by phone and accepted the intervention may have seen more need for it. Accordingly, the phone may be a beneficial mode of communication for reaching patient populations who could benefit from technological support. Our findings align with a study of a cohort of Black American women in which it was difficult to reach participants by phone; however, those who answered the phone were willing to participate [65].

We attempted to contact every patient scheduled for a telehealth visit within 2 weeks. Although our calls appeared from the FQHC, a weakness of our approach may have been the need for prior notice of upcoming calls from helpers. Thus, an additional step is to make the patients aware of the intervention before reaching out. However, steps should be in place so that every patient is informed about the call and clinicians do not unknowingly introduce disparities by predetermining who is an ideal candidate [66].

Although recent reviews have evaluated different recruitment modalities (eg, face-to-face, email, and phone) [67-69], we extend prior recruitment literature by demonstrating that people participated when someone answered the phone on the first call and that no one participated who received a voicemail. Our findings suggest that optimizing recruitment for hard-to-reach populations should include strategies that encourage potential recruits to answer the phone rather than to leave messages.

Our comparison of intervention and nonintervention participants demonstrated that we successfully recruited people who would likely benefit from helping sessions. Representation is an ongoing issue in health informatics research, and a systematic review from 2011 on consumer health informatics studies found that participation samples are predominantly White [70]. Despite this, a review of interventions to increase patient portal uptake for vulnerable populations found a predominant focus on technical training or navigation assistance but with a notable lack of sociodemographic data collection to determine whether interventions were reaching the intended populations [10]. Our tracking of sociodemographics and technical experience for intervention and nonintervention participants provide an example of how to evaluate whether equity-designed telehealth interventions reach their intended audiences. To scale up an evaluation of our equity-focused study design, we recommend extending the timeframe of the delivery of the intervention to reach sufficient power and sample size of populations often underrepresented in health informatics research.

#### Intervention Design: Sociotechnical Context

The design of our 2-week helping session intervention with patients at FQHC resulted in connection with over half (124/239, 51.9%) of the patients by phone. A California study also offered a 2-week telehealth intermediary intervention with an urban safety net that reached 67.8% (202/298) of participants [14]. Although both studies included more than half of the patients over the phone, we had far fewer (34/239, 14.2%) participants in our helping sessions than the California study (109/202, 54%) [14].

The required time for the session was a notable difference in the design of the 2 studies that may explain this difference; in our study, patients were informed that sessions would be 10-30 minutes versus 5-10 minutes for the California study [14]. This difference in time commitment may have discouraged people from participating in our study. People who already had limited available time before the pandemic may have had additional roles introduced during the COVID-19 pandemic while being in occupations that may not have allowed them to work at home [71,72]. The timing of the delivery intervention may have also been important, as most people participated within 1 to 2 days of their scheduled telehealth visit. Time has been recognized as a social determinant of health [73]; people with lower income may have less control over their time because of caregiving and community roles, precarious employment, and time involved in navigating structural barriers to care and services [73-75]. Accordingly, we recommend that future sessions investigate the ideal length and timing of equity-focused interventions while balancing potential tradeoffs between promoting uptake and intervention effectiveness.

Equity-focused telehealth studies have measured telehealth uptake disparities in large academic health care systems that have their telehealth and patient portal partially [11] or fully integrated [76], or by offering training on a single platform to a safety-net population [14]. Our intervention builds on these studies by illuminating how the lack of integration of multiple health care platforms introduces context-switching, which may increase cognitive load. In addition, our study with an FQHC demonstrates that only some clinics can afford the recommended integrated telehealth platforms with advanced telehealth features [77,78], thus reinforcing the need for ongoing, sustainable intermediary strategies to address technological disparities.

#### Intervention Process

##### How the Tasks May Have Impacted Cognitive Load

During the helping sessions, intervention participants often experienced challenges when performing complex tasks and processes. The challenges helpers noted parallel prior research on cognitive load reduction: working memory can be taxed when needing to hold verbal text (such as PINs) [24], having essential text located at the bottom of a screen [24], or context-switching across multiple programs involving a complex interaction of several elements [22,24,79]. In addition, our user-testing of the telehealth process and systems used in the FQHC (n=22) demonstrated similar cognitive load demands when completing the 3 intervention tasks. We observed the processes for logging into the patient portal, finding the telehealth consent form, and navigating between the platforms to be particularly complex and mentally demanding (Williamson and Veinot, unpublished data, September 2022).

Given that many intervention participants used cell phones during the session, completing tasks on devices with small screens may have heightened cognitive load [80-82]. Strategies to address these additional cognitive load demands suggested from previous qualitative studies with low-income populations include a preference for SMS text messaging because of its ease of use and time saving when compared with email [83,84]. Although further research is needed to address the cognitive load demands distinct from those of mobile devices [80,81], one tactic suggested by our data is that password resets may be less difficult to complete through text messaging than email.

##### Helpers’ Emergent Approaches in Targeting Cognitive Load

It has been well-established that increased cognitive load makes it difficult to learn new tasks and processes [22,24,85]. Problem-solving novel, complex tasks can further impact cognitive load by needing to draw from working memory to navigate unfamiliar pathways [22]. In addition, the negative emotions experienced when problem-solving through unique pathways on mobile devices can add to the cognitive load [82]. Our study demonstrated how the complex processes and tasks of telehealth visits might make it impossible to avoid problem-solving when navigating patient-facing technologies. However, the helpers’ technique of *building rapport to establish confidence while problem-solving* demonstrates how patients’ frustration can be alleviated when solving unfamiliar telehealth tasks. Moreover, prior research suggests that the positive emotions we aim to encourage can help reduce cognitive load [86,87] and increase motivation to learn [86,87]. The examples provided in Multimedia Appendix 6 on how and when to use these warm accompaniment techniques for cognitive load reduction can inform the design of large-scale, equity-focused technological interventions.

In our study, we found that intervention participants experienced barriers more commonly among socioeconomically disadvantaged people, including poor internet connections [14,88], small screen sizes because of mobile device use [80-82] and distractions in the physical environment [82,85]. Notably, these can increase cognitive load [21,22,80]. In addition, tasks involving processing visuospatial information versus verbal cues can have a stronger impact on negative emotions, which can tax working memory and thus increase cognitive load [87]. Helpers’ techniques of *being on the same page* and *step-by-step guidance* suggest how the auditory cues and warm accompaniment during helping sessions could help target cognitive load reduction: verbally leading people through multiple steps potentially may lessen the need for participants to focus on the visual layout of the platforms, which may be particularly frustrating when context-switching on small devices.

The impact of negative emotions when interacting with complex patient-facing technologies has broader implications, as patients may already be experiencing fear and worry from the news they may receive during their health care visit. Preliminary research suggests that negative emotions may detract from learning and decision-making and, as such, increase cognitive load [87,89]. Crucially, during a telehealth visit, dedication to a patient’s cognitive resources should not be on solving frustrating technological challenges, but on making health care decisions.

##### Impact of the Intervention on Participants

Although 15 patients had successful telehealth test runs during the helping session, analyses of the final telehealth visit modality revealed that more people switched from video to phone than vice versa. In some cases, technical problems and the unavailability of videos influenced the visit modality, even when a video visit test run was successfully completed. Provider preferences may also have shaped visit modality to an unknown degree, as previous patient portal reviews note that providers’ endorsement and perceptions of who uses technology can influence patients’ technology adoption [90,91]. Furthermore, at the time of the visit, video visits resulted in more technical problems than phone visits for patients, regardless of whether they had a helping session. This demonstrates that video visits may not always be possible, and telehealth phone visits may still be required, even when video options are available [20,92].

##### Impact on Visit Experience

Findings related to the impact of the intervention showed that intervention participants found telehealth software more difficult to learn and were less satisfied with their video visit experience than nonintervention participants. There were no significant differences in the difficulty of using technology for telehealth tasks or in self-efficacy. As this was an observational study without randomization, we postulate that these differences were rooted in the baseline differences between the groups regarding education, technology experience, and technology use frequency.

#### Design Recommendations for Future Large-Scaled Equity-Focused Studies

We found that 3 tasks were possibly too many for a single session, and the particular task of signing telehealth consent may have introduced unique elements that did not facilitate learning. Before launching a helping session intervention, we recommend using multiple types of devices and platforms to map out the elements and context switches involved in each proposed task. This process can help to structure helping sessions by identifying (1) the elements used for future applications, and thus when to focus on learning, and (2) the nonrecurring elements that tax working memory, and thus, when to introduce helping techniques to target cognitive load reduction.

The challenges encountered during the intervention suggest that making telehealth video visits feasible for patients at FQHC may require both human intermediary support and improved access to web-based technologies. The recently proposed digital inclusion-informed efforts provide a conceptual framework for designing blended strategies for future randomized equity-focused telehealth interventions [93,94] by considering affordable, robust internet-enabled devices, digital literacy training, quality technical support, and designing web-based content that encourages self-sufficiency and participation [93,94]. Recent programs that lend Wi-Fi hot spots [95], laptops [96], and smartphones [97,98] and distribute tablets to veterans with access challenges [99] offer a possible way forward in addressing the technology and internet access challenges experienced during our intervention.

### Limitations

Although we did not have a direct measure of cognitive load, we applied subjective measures of difficulty, which have previously shown a strong relationship with cognitive load [22]. Our survey results demonstrated that we successfully reached our target population; however, the low response rates to surveys and postintervention interviews indicate caution when interpreting the results. We sought diverse representations of participants by having helping sessions delivered by students who represented diverse cultures, races, ethnicities, and genders; however, there needed to be more representation of some demographics. More women than men participated in the intervention, and older adults were distinctly missing, as no participant over the age of 61 participated in the intervention. James and Harville’s [75] study on Black men’s participation in research noted lack of time, privacy concerns, and mistrust of researchers as common barriers [75]. A study on encouraging recruitment of low-income older adults also emphasized the importance of establishing trusting relationships and recommended offering more than one recruitment strategy [100]. The urgency of our telehealth intervention in response to the COVID-19 pandemic did not make it feasible to offer multiple forms of long-term recruitment that encouraged the establishment of trust before recruitment. However, the helpers’ intervention technique for building rapport may have instilled the participants’ confidence and trust during the session.

### Conclusions

This equity-focused pilot study on preparing patients at FQHC for an upcoming video telehealth visit builds on literature regarding telehealth access promotion strategies by targeting cognitive load through the *warm accompaniment of intermediaries.* We offer 3 possible techniques for targeting cognitive load when navigating complex patient-facing technologies: *providing step-by-step guidance for configuring and learning*, *building rapport to establish confidence while problem-solving* and *being on the same page to counter informational distractions*. However, the limited number of helping session participants illustrates the ongoing challenges of video visit access for patients at FQHC and further demonstrates the need for research regarding sustainable and equitable digital health strategies. Although cognitive load reduction may be a valuable focus, patients at FQHC may require long-term assistance and improved technology and internet access to make telehealth video visits feasible, even with helping sessions.

## Appendix

Multimedia Appendix 1 Helping documents for intervention participants.

Multimedia Appendix 2 Script for helping sessions.

Multimedia Appendix 3 Wiki document for helpers.

Multimedia Appendix 4 Structured forms used by helpers.

Multimedia Appendix 5 Tracking of decline to participate.

Multimedia Appendix 6 Examples of 3 helping strategies.

Multimedia Appendix 7 Summary statistics phone versus video telehealth visits.

## Abbreviations

EHR: electronic health record
FQHC: Federally Qualified Health Center
