# Supplementary material for: Targeting Patients’ Cognitive Load for Telehealth Video Visits Through Student-Delivered Helping Sessions at a United States Federally Qualified Health Center: Equity-Focused, Mixed Methods Pilot Intervention Study
Source: J Med Internet Res. 2023 Feb 1;25:e42586. doi: 10.2196/42586 (PMC9897309; doi:10.2196/42586)

Multimedia Appendix 1: Helping Documents for Intervention Participants

Two helping documents were created for the intervention participants. The first provided patient portal information on: 1) what a patient portal is; 2) patient portal features; 3) and steps for registering for a patient portal. The second was a telehealth guide that was divided into three sections: 1) pre-visit checklist; 2) day of video visit checklist; and 3) 15 minutes before appointment. Below is a sample page from the Telehealth Helping Guide.

DAY OF VIDEO VISIT CHECKLIST

- ***Make sure your phone or laptop is fully charged***
  Keep your charger near you if needed
- ***Find a private location for your telehealth video visit***
  You can increase your privacy by being in a room where you can shut the door and by using earphones.
- ***Turn off other devices***
  Turning off other devices on the same wifi connection can help better your internet speed.
- ***Move to where the internet signal is strongest***
  If you have your own router you should move closer to it.
- ***If you have a WiFi router, you can make your internet signal stronger by:***
  - Moving closer to it
  - Moving anything that might be blocking it
- ***If you are using a computer, use an ethernet cable to connect to the router***
  Using an ethernet cable will give you the most stable connection.

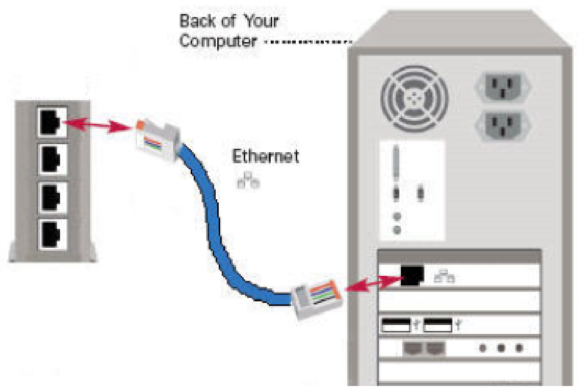

Supplement: Multimedia Appendix 1 [file jmir_v25i1e42586_app1.pdf]
