# Supplementary material for: Targeting Patients’ Cognitive Load for Telehealth Video Visits Through Student-Delivered Helping Sessions at a United States Federally Qualified Health Center: Equity-Focused, Mixed Methods Pilot Intervention Study
Source: J Med Internet Res. 2023 Feb 1;25:e42586. doi: 10.2196/42586 (PMC9897309; doi:10.2196/42586)
Supplement: Multimedia Appendix 2 [file jmir_v25i1e42586_app2.pdf]

Multimedia Appendix 2: Script for Helping Sessions

Reaching out: Needs assessment

[Note that in keeping with the emergent nature of the dialogue with patients, the wording of the questions to be asked may change, as may some of the technical topics covered]

Phone support - Before patients' appointments, Intermediary, a technology helper, will call patient to offer assistance

Role and Resources needed:

- **Set up:** Call the patient
  - Call patients using Telehealth platform #2 online telehealth platform with accounts provided by the clinic as this allows the call to be displayed as from the clinic
  - Before the call, determine patient's name and appointment date/time
- **Navigate/Guide:** Remind patient about resources available to them and send them navigation guide via the desired method (email, patient portal)
  - Have wiki for intermediaries open
- **Remember your Role as a technology helper. This means you should:**
  - Help the patient
  - Be an active listener
  - Answer questions
  - Be professional

Introduction: *Introduce yourself and the reason for your call*

- Warm greeting
- State your name, the connection and your role
- Ask to speak to patient, confirm you are speaking to the patient
- State reason for call
- Offer your assistance

Sample Introduction: You may change this up so it sounds more authentic to you
Hello, this [say your name], I am a technology helper working with [name of clinic] and the University of Michigan. May I speak with [insert patients first name here]?

Hello, [patients name]. Before I proceed, I need to make sure I'm talking to the right [patient's name]. What is your last name?

[If a video visit] I see here you have a [video] appointment coming up soon with Dr. [name] at [Date and Time]. I am calling to help you prepare for your appointment. I can answer any questions about setting up your device for the ***video call*** *or navigating your* ***patient portal,*** *which you will need for your video visit.*

[If a phone visit] I see here that you have a telephone appointment coming up soon with Dr. [name] at [Date and Time]. I am calling to let you know that it is an option to have your visit via video rather than just telephone. In a video visit, you will be able to see and hear Dr. [name] and [they/he/she] will be able to see you. Are you interested in having a video visit instead of a telephone visit if you can?

[If no] What makes you not be interested in a video visit? [record answers verbatim]

[If yes, proceed the script below] I can help you prepare for your appointment. I can answer any questions about setting up your device for the ***video call*** *or navigating your* ***patient portal,*** *which you will need for your video visit.*

Do you have any questions for me before we begin?

- If yes, answer their question.
- If not, ask them if they have a few minutes to go through a readiness checklist?
  - *We want to go through this checklist to make sure your appointment is hassle free.*

Readiness checklist (Need assessment): *Checklist to confirm patients readability for appointment*

- Use appreciative inquiry strategies
- Thank them for their time.
- This will take about 10 - 30 minutes of their time

1. Ability to receive messages (*What way do you like to receive messages about your appointment?)*
   - ❑ Email
   - ❑ Text message
   - ❑ Patient portal
2. Device
   - ❑ Phone/ Laptop /tablet
     - Device age, features, and capabilities
       - *How long have you had your device for?*
       - *Tell me about your experience with your device and camera?*
       - *What do you find is the best way you get internet access on your device?*
       - *What internet browser do you like to use on your device?*
     - Familiarity with device
       - *Have you used it before for a video call, what was your best experience with it like? (Can you tell me about it?)*
     - Device Suggestions
       - *Charge your phone before your appointment*
       - *Make sure volume is up*
       - *Use headphones*
       - *Strong wifi connection*
3. Location and connectivity (*On the day of your visit, we want to give you a few recommendations to have a successful video call*)
   - ❑ A quiet and private place to receive your phone call.

- *This is important because this is an actual doctor's [or healthcare provider] visit. So you and your doctor will discuss everything you would normally discuss at a medical visit. You want to make sure you are in a private place where people cannot overhear your conversation. And you want to be able to clearly hear your doctor [or healthcare provider].*
- *Do you have any questions or concerns about finding a quiet or private place to have your* ***video call****?*

❑ Adequate internet access

- *Walk through how to check Internet access.*
- *Do you have any questions or concerns surrounding gaining or checking your internet access?*

4. Telehealth consent (*The next step is a consent. Do you have a patient portal?*)

☐ Set up for them in the Patient portal EHR if necessary

☐ Lead through telehealth consent

- Quick View/Communicator/Log in to Portal as Patient
- Click on manage appointments
- Click on manage my appointments
- Click see more on the bottom arrow
- Click on 2 forms to complete
- Click here to read the consent document.
- Select English if wanting to switch from Spanish to English
    - Click on here link to review document
    - Click on box to agree that you have read and understand the information
    - Enter name, date and relationship
    - Click submit

5. What to expect the day of your visit.

- *You will receive a call on the day of your visit reminding you about it, and the steps to take for your appointment. Are you interested in hearing about it now? (Yes/No)*
    - *Great! It is a 4 step process.*
        - ➢ ***Click****! Click the link.*
        - ➢ ***Name!*** *Type your name on the welcome screen*
        - ➢ ***Check*** *your picture and sound.*
        - ➢ ***Wait!*** *Wait for the doctor to come on.*
    - *You will receive the steps again on the day of your visit.*

6. Check the link with them.

❑ Send Link

- *We will like to send you a link to make sure your device is working properly.*
- *Are you interested in going through the process of connecting to a visit now? (Yes/Not right now in ___ minutes)*

Conclusion: Summarize and thank patient for time

- Summarize the process
- Ask patient if they have any further questions
- Tell patient about follow up opportunities
- Thank patient for their time

Sample Conclusion

Today we accomplished the following …

Do you have any additional questions?

It was great chatting with you, [say patients name]. I hope all your questions were answered.

The team and I are available up until your appointment to answer any questions you may have. Feel free to reach out to us at [ ], there is a team of available persons here to help you with all your ehealth technology set up and navigation needs.

Thank you for your time. I hope your ***video call*** *appointment goes well. Have a great day!*

Follow-Up by Patient

Patients may follow up or call intermediaries to ask for help or ask more questions

When to expect call:

- Anytime, day of, hour before.

What to expect:

- Questions about set up
  - Haven't receive their link yet
  - Link not working
  - Phone not working
  - Connectivity issue
- Questions about navigation
  - where to find [blank] on patient portal
  - how to do [blank]

What resources are available to you to answer questions

- Troubleshooting page
- Navigation guide
- Your fellow intermediaries

How to respond: Remember responsibilities

- Greet patient
- Assess the urgency (amount of time they have to solve this issue)
- Reiterate question
- Answer questions
  - Ask for help if you are unable to answer question
  - Be sure to describe the question in intermediary *troubleshooting guide*
- Ask if all questions have been answered
- Offer additional resources
- Say good bye

Sample response

Hello, [Callers name].

If I understand you correctly you want help solving/with [problem].

What methods have you already tried to solve this problem?

I am sorry those ways did not work for you.

When/what time is your appointment?

(Great we have time/ we don't have much time) Why don't we try [go through a list of possible solutions]?

Thank you for calling today, I hope all your questions are answered. As always available to use for assistance, and we would like to offer you an additional resource [navigation guide] for your benefit next time. Thank you for calling us I hope you have a great day.
