# Supplementary material for: Targeting Patients’ Cognitive Load for Telehealth Video Visits Through Student-Delivered Helping Sessions at a United States Federally Qualified Health Center: Equity-Focused, Mixed Methods Pilot Intervention Study
Source: J Med Internet Res. 2023 Feb 1;25:e42586. doi: 10.2196/42586 (PMC9897309; doi:10.2196/42586)
Supplement: Multimedia Appendix 3 [file jmir_v25i1e42586_app3.pdf]

Multimedia Appendix 3: Wiki Document for Helpers

The Wiki document was a helping guide for student helpers that had tabs for setting up Wi-Fi, Audio/Video and Language preferences. Below is a sample of the Wi-Fi Wiki Page.

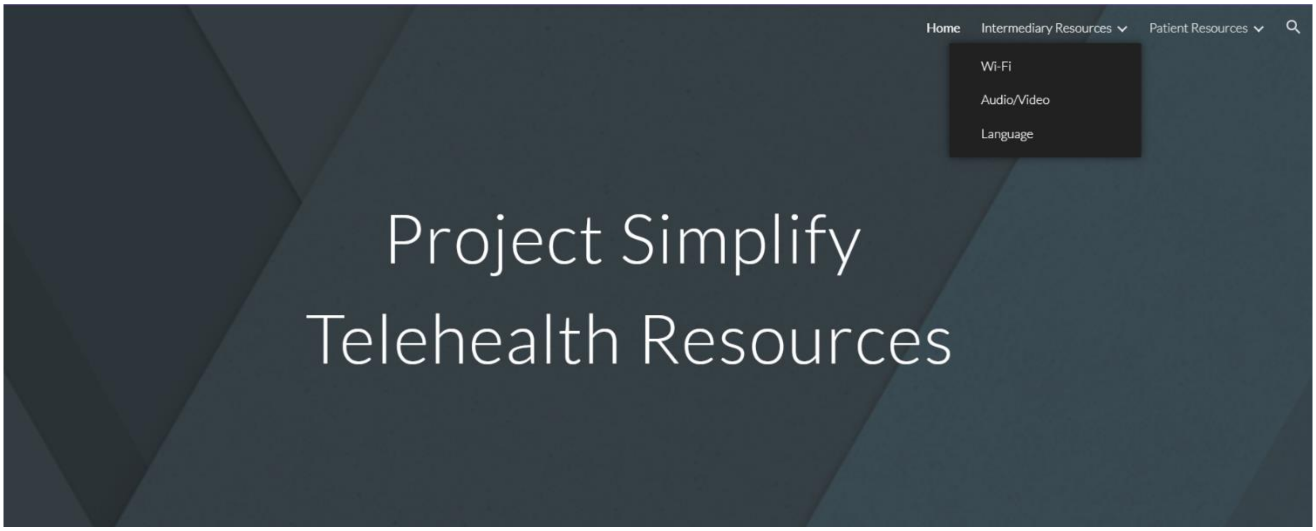

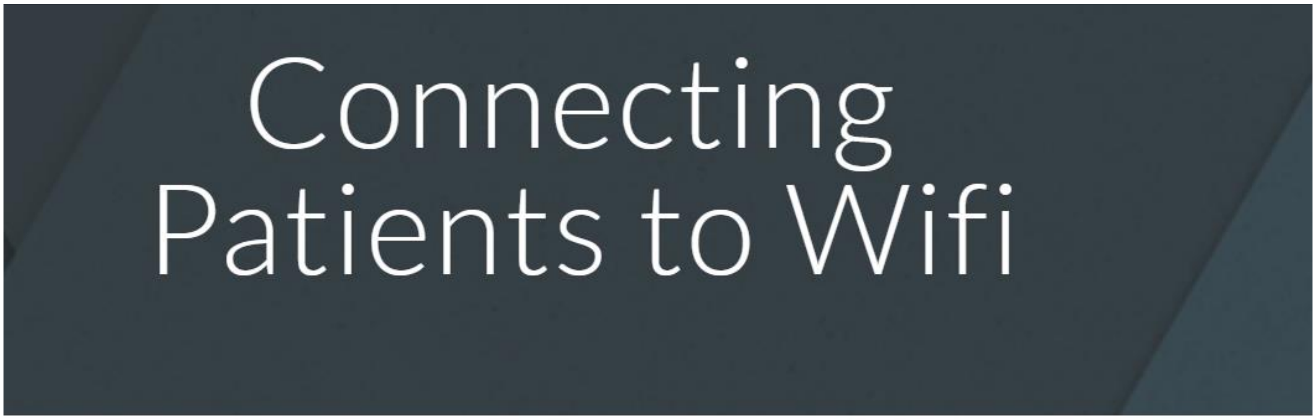

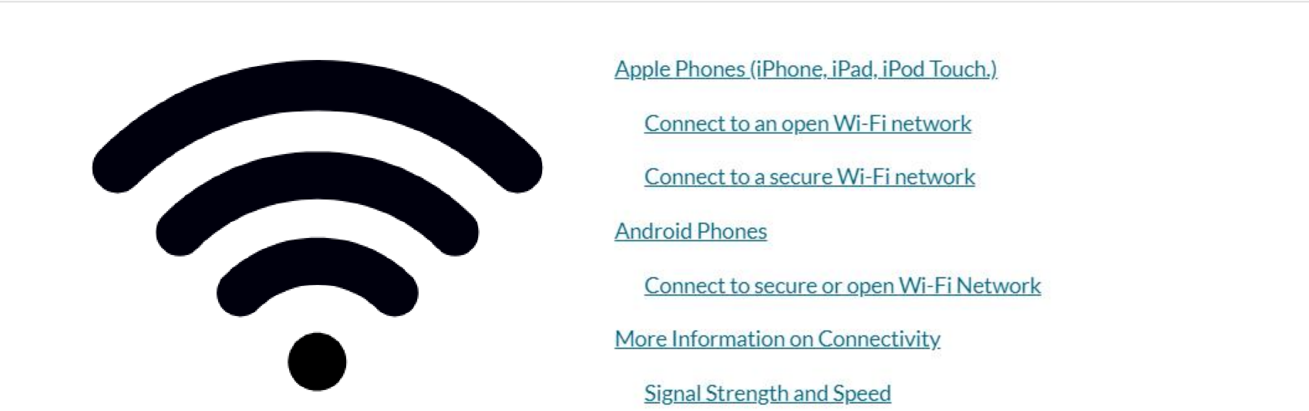

Apple Phones (iPhone, iPad, iPod Touch.)

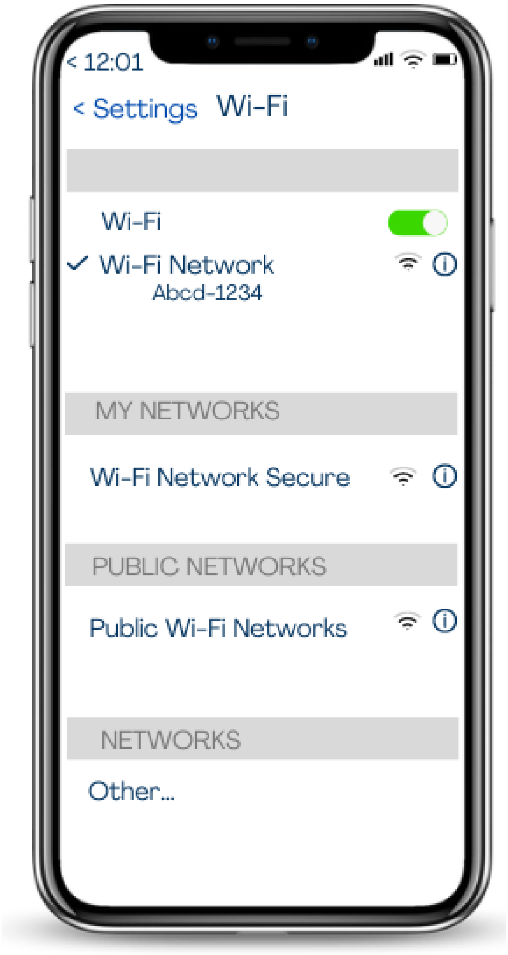

Connect to an open Wi-Fi network

1. From your Home screen, go to Settings > Wi-Fi.
2. Turn on Wi-Fi. Your device will automatically search for available Wi-Fi networks.
3. Tap the name of the Wi-Fi network that you want to join.

With iOS 13 and iPadOS, you might see these network options:

- My Networks: Wi-Fi networks that you've connected with before.
- Public Networks: Available Wi-Fi networks that you've never connected with before.

After you join a network, you'll see next to the network and in the upper-left corner of your display or the upper-right corner on an iPhone X and later.

More Information on Connectivity

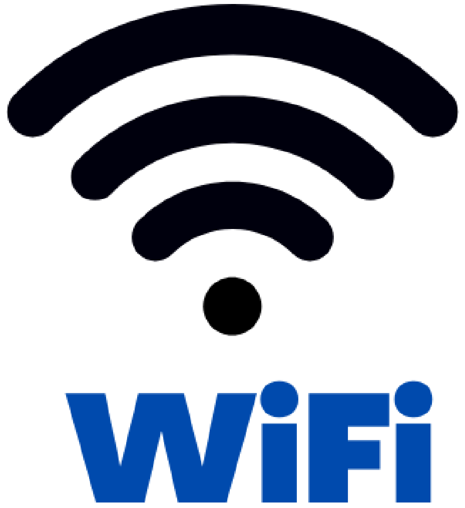

Signal Strength and Speed

The network's ***signal strength*** is on the Wi-Fi icon . A fuller icon means a stronger signal. ***Signal speed*** can change with signal strength.

- **Very Fast**: You can stream very high-quality videos.
- **Fast**: You can stream most high-definition (HD) videos and make video calls.
- **OK**: You can read webpages, use social media, and stream music and standard-definition (SD) videos.
- **Slow**: You can send and receive emails and texts. Images appear slowly.
