# Supplementary material for: Targeting Patients’ Cognitive Load for Telehealth Video Visits Through Student-Delivered Helping Sessions at a United States Federally Qualified Health Center: Equity-Focused, Mixed Methods Pilot Intervention Study
Source: J Med Internet Res. 2023 Feb 1;25:e42586. doi: 10.2196/42586 (PMC9897309; doi:10.2196/42586)
Supplement: Multimedia Appendix 4 [file jmir_v25i1e42586_app4.pdf]

Multimedia Appendix 4: Structured Forms Used by Helpers

Form 1: Daily Reflection Notes

At the end of each day conducting the intervention, the technology helper should take 30 min-1 hour reflecting on their experiences during that day. The prompts are here to help you reflect on these areas, but feel free to include information/details that are not requested in these prompts. Save this document as Lastname MM.DD.YY

Helper Name: ____________

Date: _________________

Experience with Patients

1. Step by step, can you walk through an interaction that was particularly positive today?
2. Step by step, can you walk through an interaction that did not go as planned today?
3. How did you feel when interacting with patients today? What made you feel that way?

Communication

1. How did you try to build rapport with patients today? What worked well? What didn't work well?
2. How did you make sure you fully understood the patient's problem(s)?
3. Were the patients able to understand your instructions during your sessions? What were approaches you took to explain things clearly?
4. Was anything particularly difficult to explain to the patients? If so, what was it, and what was difficult about it?

Problem solving

1. Were there any approaches to providing support that *didn't* work well? If so, describe…
2. Were there any approaches to providing support that *did* work well? If so, describe…

Complexity reduction

1. In your visits today, did patients struggle to understand any part of telehealth visits? How did you help them gain understanding?
2. In your visits today, did patients get stuck in any part of telehealth visits? How did you help them get unstuck?
3. Do you think your interactions with patients today helped to simplify telehealth for them? If so, how?

Conclusion

1. What was the most important interaction you had with a patient today? What made it important?

Form 2: Structured Observations

Intermediary Visit Observation Sheet
[to be completed by the technology helper during and/or after each helping session- save as Lastname.MM.DD.YY.ParticipantID.docx
Participant ID ______

Date: _________ Provider: _________Clinic: ____ _________

Scheduled Visit Date: ________ Scheduled Visit Time: ___

Helper Name: ________________

Start time: __________ End time: _________________

**BACKGROUND**	❑
**Visit Modality**	❑ **Telephone visit**<br>❑ **Video Visit with Telehealth platform #1**<br>❑ **Video Visit with Telehealth platform #2**
(Telephone visit) **Is patient interested in video visits?**	
(Telephone visit, If patient is not interested in video visits) **What makes you not be interested in video visits?** (write response verbatim)	
**HELPING SESSION**	
**Patient Information:**	Gender:<br>Age estimate:<br>Technology used for intermediary call:<br>Technology used for telehealth visits:
**Location of issue/guidance**	❑ **Internet Connectivity**<br>❑ **Patient device**<br>❑ **Patient portal**<br>❑ **Video conferencing tool (Telehealth platform #1 or Telehealth platform #2)**<br>❑ **Other _No issues just curious____**
**Communication with patient**	
Describe the patient's initial demeanor.	
How did the patient describe their concerns or issues, if any?	

(If relevant) What did you do to find out more about the problem?	
Are there places where they got confused but did not articulate? What were they?	
How did the patient respond to your help?	
If you had any technical issues with solving the patient's problem, please describe them here.	
**Guidance provided**	
Was the issue resolved? If so, describe how the problem was solved.	
Did you show the patient how to do something? If so, what?	
**Notes**	
