# Supplementary material for: Targeting Patients’ Cognitive Load for Telehealth Video Visits Through Student-Delivered Helping Sessions at a United States Federally Qualified Health Center: Equity-Focused, Mixed Methods Pilot Intervention Study
Source: J Med Internet Res. 2023 Feb 1;25:e42586. doi: 10.2196/42586 (PMC9897309; doi:10.2196/42586)
Supplement: Multimedia Appendix 5 [file jmir_v25i1e42586_app5.pdf]

Multimedia Appendix 5: Tracking of Decline to Participate

Reason	Number of People
**Lack of Interest, Need or Time**	
Already comfortable with using telehealth and do not need help	9
Not Interested	9
Busy	9
Already have the required help from the clinic	1
**Technical Reasons**	
Not having phone capabilities for video calls	3
More comfortable with using the phone	3
Not feeling comfortable with their technical skills	2
Concerned about fees being charged to their phone	1
**Health Related**	
Poor health status	5
In care facility	2
**Scheduling**	
Had forgotten about upcoming visit	5
Had rescheduled visit for earlier in the week	1
**Other Reasons**	
Distrustful of call	2
**Reason not Provided**	
Not reached again after initial phone contact	20
Refused without providing an answer	9
Hung-up	6
Not proficient in English or Spanish	2
Poor phone connection, could not hear	1
