# Supplementary material for: Targeting Patients’ Cognitive Load for Telehealth Video Visits Through Student-Delivered Helping Sessions at a United States Federally Qualified Health Center: Equity-Focused, Mixed Methods Pilot Intervention Study
Source: J Med Internet Res. 2023 Feb 1;25:e42586. doi: 10.2196/42586 (PMC9897309; doi:10.2196/42586)
Supplement: Multimedia Appendix 6 [file jmir_v25i1e42586_app6.pdf]

Multimedia Appendix 6: Examples of 3 Helping Strategies

**Providing Step-by-Step Guidance for Configuring and Learning**			
	**When**	**How**	**Example**
	Switching between platforms (i.e., context switching)	Helper assists in remembering temporary information, such as security codes	*I told him that if he wanted help remembering the security code to get into his account, he could tell it to me, and I could repeat it back to him when he went to plug the number in for verification. This proved to be helpful as he forgot the number when we left the text message*".
	During configuration and set-up	Pause and check-in at each critical stage	"*I walked him through the steps again to connect to [the video platform] and checked in after each step. For example, did he receive the link via text?; was he able to click on the link?; did the link open?; did the page load?; was he able to see a box to type in his name?; could he press submit to go to the next page? Eventually we figured out the blockage was that his phone said "another app is accessing the camera and or microphone". I told him we'd try disconnecting the phone call and see if that allowed him to get onto [the video platform] and it did. I told him to make sure all his other apps were closed out before his appointment*".
**Building Rapport to Establish Confidence while Problem-Solving**			
	**When**	**How**	**Example**
	Unexpected moments that take helpers "off-script	Creating more natural conversations that connect with each other and address patient's needs	I "*flubbed the lines*" with the participant, the two "*had a good laugh,*" and from that point on the session "*felt more conversational.*"<br><br>During another session that began in English, the participant picked up cues from the helper that they were both Spanish-speaking, and they switched to speaking "*Spanglish,*" making the session more relaxed.<br><br>Another session demonstrated added value when the helper spent a bit more time addressing the participant's request by providing guidance on how to send a message to their healthcare provider in the patient portal.
	Encountering unfamiliar tasks	Sharing emotions when figuring it out together	Helpers saw that the helping session had not only "*built [participants'] confidence in knowing what to expect*" but that the sessions were also "*a confidence builder for me [the helper].*"

			During the interviews, one participant noted that they and the helper were "*just trying to figure it [the telehealth consent] out on both ends. I think [the helper] was just as confused as I was.*"<br><br>While another participant also noted how they had "*doubts*" and were "*a little confused about how to open the telehealth page [video visit platform], but then [it] became easier.*"<br><br>Joy was also shared when difficulties were successfully addressed. This is demonstrated through a helpers' reflection note, "*Finally SUCCESS!! We were both so happy*".<br><br>"*it felt like a win, especially because neither of them was confident they would be able to do it.*"
**Being on the Same Page to Counter Informational Distractions**			
	**When**	**How**	**Example**
	Seeing different views due to different platforms	Use the same type of platform	After one session, the helper noted how they *"felt that we weren't on the same page in terms of what I thought they were seeing and what they were actually seeing since they were on the mobile version."* After encountering this issue early in the intervention, helpers got on the same page by learning to switch to a mobile phone view during the intervention.
	Informational distractions	Check in to see if the terms being used are understood	A helper related how they, "*talk[ed] back [to the patient to]… make sure we meant same thing*".<br><br>In contrast, the introduction of unfamiliar technical terms demonstrated that unfamiliar words can disrupt processes for "*being on the same page*". This issue was further shown during the interviews when a participant did not know how to respond to questions about using "*the patient portal*" and responded with confusion "*the what?*", and was then able to answer the question when asked whether they use "*the online web site… for test rests and upcoming visits*".
