# Supplementary material for: Targeting Patients’ Cognitive Load for Telehealth Video Visits Through Student-Delivered Helping Sessions at a United States Federally Qualified Health Center: Equity-Focused, Mixed Methods Pilot Intervention Study
Source: J Med Internet Res. 2023 Feb 1;25:e42586. doi: 10.2196/42586 (PMC9897309; doi:10.2196/42586)
Supplement: Multimedia Appendix 7 [file jmir_v25i1e42586_app7.pdf]

Multimedia Appendix 7: Summary Statistics Phone versus Video Telehealth Visits

Table A: Phone Visits versus Video Visits

Variable	**Overall** n= 128[1]	**No Intervention** n = 113[1]	**Intervention** n = 15[1]	**p-value**[2,3]
**Age** (years)	44.4 (13.7)	44.4 (13.9)	43.9 (12.4)	0.90
**Visit Type**				0.94
Phone Visit (audio only)	67 / 128 (52%)	59/ 113 (52%)	8 / 15 (53%)	
Video Visit	61 / 128 (48%)	54 / 113 (48%)	7 / 15 (47%)	

[1] n / N (%); Mean (SD)
[2] Pearson's Chi-squared test; Wilcoxon rank sum test; Fisher's exact test
[3] Bonferroni correction not used. Sample size is accounted for within tests.

Table B: Phone Visits: Summary Statistics for FHQC Telehealth Survey

**Variable**	**Overall** n = 67[1]	**No Intervention** n = 59[1]	**Intervention** n = 8[1]	**p-value**[2]
**Any Phone Problems**	3 / 67(4.5%)	3 / 59 (5.1%)	0 / 8 (0%)	0.99
**The call dropped and we tried again.**	3 / 67 (4.5%)	3 / 59(5.1%)	0 / 8 (0%)	0.99
**Had Sound Problems**	1 / 63 (1.6%)	1 /56 (1.8%)	0 / 7 (0%)	0.99
(Missing)	4	3	1	

[1] n / N (%)
[2] Pearson's Chi-squared test; Fisher's exact test

Table C: Phone Visits: Summary Statistics for Types of Problems Encountered

Problems	No Intervention	Intervention
**The call dropped, and we had to try again.**	3	0
**How many times did the call drop?**	1 (2x); 3 (1x)	0
**There were problems with the sound during my call: "The call kept on freezing and I couldn't hear."**	1	0

Table D: Video Visits: Summary Statistics for FQHC Telehealth Survey

Variable	Overall n = 61[1]	No Intervention n = 54[1]	Intervention n = 7[1]	p-value[2]
**Any Video Problems**	7 / 61 (11%)	6 / 54 (11%)	1 / 7 (14%)	0.99
**Because of problems, we switched to a phone visit.**	4 / 59 (6.8%)	3 / 53 (5.7%)	1 / 6 (17%)	0.36
(Missing)	2	1	1	
**Had Sound Problems**	2 / 59(3.4%)	2 / 52 (3.8%)	0 / 7 (0%)	0.99
(Missing)	2	2	0	

[1] n / N (%)
[2] Fisher's exact test

Table E: Video Visits: Summary Statistics for Types of Problems Encountered

Problems*	No Intervention	Intervention
**Any Problems during my video visit.**	7	0
**There were problems with picture during my video visit.**	3	0
**I had problems setting up the video visit software on my device (phone, tablet, or computer).**	2	0
**I couldn’t hear my healthcare provider or My healthcare provider couldn’t hear me.**	2	0
**Our video visit wasn't working and we had to try again.**	2	1
**The picture froze.**	2	0
**I couldn’t see my healthcare provider or My healthcare provider couldn’t see me.**	1	0
**“It just wasn’t the best quality.”**	1	0

*multiple responses possible.
